# Extracellular matrix density regulates the formation of tumour spheroids through cell migration

**DOI:** 10.1371/journal.pcbi.1008764

**Published:** 2021-02-26

**Authors:** Inês G. Gonçalves, Jose Manuel Garcia-Aznar

**Affiliations:** Multiscale in Mechanical and Biological Engineering, Aragon Institute of Engineering Research, Mechanical Engineering Department, University of Zaragoza, Zaragoza, Spain; Oxford, UNITED KINGDOM

## Abstract

In this work, we show how the mechanical properties of the cellular microenvironment modulate the growth of tumour spheroids. Based on the composition of the extracellular matrix, its stiffness and architecture can significantly vary, subsequently influencing cell movement and tumour growth. However, it is still unclear exactly how both of these processes are regulated by the matrix composition. Here, we present a centre-based computational model that describes how collagen density, which modulates the steric hindrance properties of the matrix, governs individual cell migration and, consequently, leads to the formation of multicellular clusters of varying size. The model was calibrated using previously published experimental data, replicating a set of experiments in which cells were seeded in collagen matrices of different collagen densities, hence producing distinct mechanical properties. At an initial stage, we tracked individual cell trajectories and speeds. Subsequently, the formation of multicellular clusters was also analysed by quantifying their size. Overall, the results showed that our model could accurately replicate what was previously seen experimentally. Specifically, we showed that cells seeded in matrices with low collagen density tended to migrate more. Accordingly, cells strayed away from their original cluster and thus promoted the formation of small structures. In contrast, we also showed that high collagen densities hindered cell migration and produced multicellular clusters with increased volume. In conclusion, this model not only establishes a relation between matrix density and individual cell migration but also showcases how migration, or its inhibition, modulates tumour growth.

## Author summary

Multicellular organisms are composed of cells found in a scaffold known as the extracellular matrix, which interacts with cells. There is still a need to understand how the properties of this matrix, namely, its mechanical properties, regulate the organization of cellular systems. However, recent works have proven the relevance of the matrix, with a particular emphasis in tumour biology studies. Furthermore, to accelerate and reduce the costs of these studies, several computational frameworks have been presented to simulate the collective behaviour of the matrix. Hence, in this work, we introduce a model based on experimental data, which highlights the role of the mechanical properties of the matrix in individual and collective cell migration. We clearly show how the extracellular matrix induces the formation of large tumour clusters. Moreover, the model that we present accurately describes general trends of the experimental results used for model calibration; the model also has the potential to be extended to study matrices with different properties and different cell lines.

## Introduction

The extracellular matrix (ECM) is the non-cellular component present in all tissues, which not only serves as a physical scaffold that provides support to cells but also interacts with them and mediates their biological functions [1, 2]. The ECM is mainly composed of water, proteins, such as collagen, elastin and fibronectin, and polysaccharides, but the quantities at which these components are present vary significantly based on the tissue. In fact, the characteristic mechanical properties of tissues arise from the particular composition of their ECM [3, 4].

Interestingly, in recent years, more focus has been given to the interplay between the mechanical properties of the cellular microenvironment and the emergent cell behaviour, as more studies have revealed that cells sense and respond to these characteristics [5, 6]. Matrix stiffness, which characterizes the matrix’s resistance to deformation in response to applied forces, has been extensively studied as a regulator of biological processes [7–9], and cell motility in particular [10–13]. For instance, studies have shown that the matrix stiffness may influence the direction of both cell movement, directing the cells along stiffness gradients [12], and cell speed, with stiffer matrices producing higher cell velocity values [13]. Nonetheless, the majority of these works relate to 2D conditions and may not apply to 3D conditions.

In 3D configurations, stiffness values arise from structural changes that also affect the matrix architecture, which regulates migration by itself [14]. Specifically, matrices with a higher fibre density may be stiffer, but they also present smaller pore sizes, which regulate the confinement levels. Confined microenvironments are commonly associated with restrained cell motility, as cells become unable to squeeze through the matrix to continue moving [15]. Consequently, the complexity of 3D cultures makes it increasingly difficult to disassociate the effects induced by matrix stiffness from those produced by the matrix architecture.

Taking into account that the nature and composition of the ECM are quite hard to replicate *in vitro*, experimental studies conducted with 3D scaffolds have been helpful to improve the understanding of how the ECM regulates 3D cell migration through simplified models [16–19]. In particular, hydrogels mainly composed of collagen, the most abundant substance of the natural ECM components [1], are commonly used in experimental settings. Among other benefits, collagen matrices offer versatility and enable the possibility to produce matrices with different mechanical properties based on their composition and preparation procedures [20]. For example, collagen concentrations can be altered or changes in pH can be implemented to modify the different matrix properties [21].

Understanding cell motility and its dependence on the physical properties of the ECM is of particular interest in cancer biology. The mechanical properties of the tumour microenvironment differ from those found in normal tissues as a result of the tumour cells’ ability to sense and modify their environment [2, 22, 23]. The invasion of surrounding tissues by tumour cells, a process known as metastasis, represents a critical step in tumour development and leads to a significant decrease in survival rates [22, 24]. Several previous studies have aimed to assess the pathophysiological processes of primary tumours and explain how the mechanical properties of the ECM induce and guide metastasis [11, 25–27]. Nevertheless, a particular area of study that has yet to be understood is related to metastatic colonization, i.e., how metastatic cells adapt to survive in a new tissue [28].

Although the large majority of circulating tumour cells perish before they are able to produce a secondary tumour, some can invade new tissues and replicate [28, 29]. In the last few years, studies have shown that this ability to survive and proliferate in new tissues depends not only on cells but also on the biophysical microenvironment of the metastatic niche and the cell-matrix interactions [30]. Thus, it is of high importance to understand and characterize how tumour cells respond to different compositions of the microenvironment, to identify the causes that may induce a more invasive phenotype and to develop therapeutic strategies that hinder this behaviour [28, 31].

Several studies have studied tumour cells seeded in 3D scaffolds to replicate the initial stages of growth in avascular tumours [19], creating a setting that can also reflect the initial stages of metastatic colonization in new tissue. There are several available techniques used to produce multicellular structures from tumour cells [32]. Tumour spheroids, for example, can be formed by growing tumour cells in solutions or non-adhesive substrates and can later be introduced in 3D scaffolds [33]. However, individual cells may also be directly seeded in 3D matrices or microfluidic devices to understand how cells organize based on the matrix characteristics [34, 35]. Despite these advances and the various available experimental setups, it is still unclear how the mechanical properties of 3D scaffolds modulate tumour cell behaviour.

On the one hand, it is well established that matrices of higher density suppress growth by exerting compressive forces on cells, hence producing smaller tumours than those grown in matrices with low densities [36–38]. Consequently, the tumour size tends to decrease as the density of the matrix increases. In general, these experiments have used low-porosity matrices. On the other hand, experimental studies have also shown that matrices with higher density tend to limit cell movement as cells are unable to migrate through the matrix due to steric hindrance [10, 39–41]. In turn, this situation promotes individual cell migration in matrices composed of lower collagen concentrations, causing cells to stray from their original cluster, which can subsequently affect the tumour’s size [39, 42, 43].

We highlight the work presented in [39], which aimed to study tumour cells in a microfluidic setup to characterize how the collagen density of matrices affects cell organization and tumour growth from single cells seeded in those matrices. Through the use of a microfluidic experimental configuration, the authors were able to study single-cell migration, and the development of multicellular structures, as two distinct processes. Interestingly, the authors concluded that the effect of collagen density on cell migration was a key regulator in tumour spheroid formation. Whereas the matrices with low concentrations of collagen enabled cells to migrate more freely, the increased motility produced sparser and smaller clusters. In contrast, the restrained motility of cells seeded in matrices of higher collagen density resulted in larger spheroids. Accordingly, it can be stipulated that even though the invasive capability is low, cell surveillance is enhanced by denser matrices, as cells aggregate and form stable structures that evolve into secondary tumours [44, 45].

Despite the advances made regarding the experimental settings, there are still disadvantages, such as increased costs and long time scales. Accordingly, to facilitate the study of some of these biological issues, advances have been made in computational modelling to create models and simulation frameworks that can replicate experimental settings while overcoming the disadvantages. Currently, multiple computer-based frameworks have been implemented to study tumour cells and their interactions with the surrounding microenvironment, as reviewed in more detail in [46, 47]. Furthermore, the implementation can rely on continuum [48], discrete [49], or hybrid approaches [50]. Nonetheless, the adequacy highly depends on the subject of study.

Given these considerations, here, we present a model extension of the typical models developed in PhysiCell (an open-source modelling framework) in which the effect of the ECM density on cell motility and, consequently, on tumour growth is introduced [51]. We aimed to build a model that was able to capture the general tendencies presented in [39] while still having a low computational cost and flexibility to adapt to other experimental setups. Accordingly, we used the previously published results in [39] to characterize how the collagen density of the matrix restrains cell motility, without overfitting the model to the experimental data. Despite the simplicity of our model, we still obtained an accurate depiction of how the density of the matrix influences cell movement. Furthermore, at a multicellular level, our study replicates how the cells’ ability to migrate modulates tumour growth, depending on the steric hindrance properties of the ECM.

## Materials and methods

### Model design

In this study, we aimed to replicate the experimental results found in [39], namely, those regarding the role of collagen concentration on cell motility and, consequently, on tumour growth. The premise of this previous work was to seed individual cancer cells of a non-small cell lung cancer (NSCLC) cell line in collagen matrices of varying concentrations and to assess how the collagen density modulates cell behaviour. Specifically, [39] primarily introduced an experimental setup that enabled the study of single-cell motility by tracking individual cell trajectories for 24 hours and computing metrics such as the distance travelled by cells and their effective and mean speeds.

**Fig 1:**
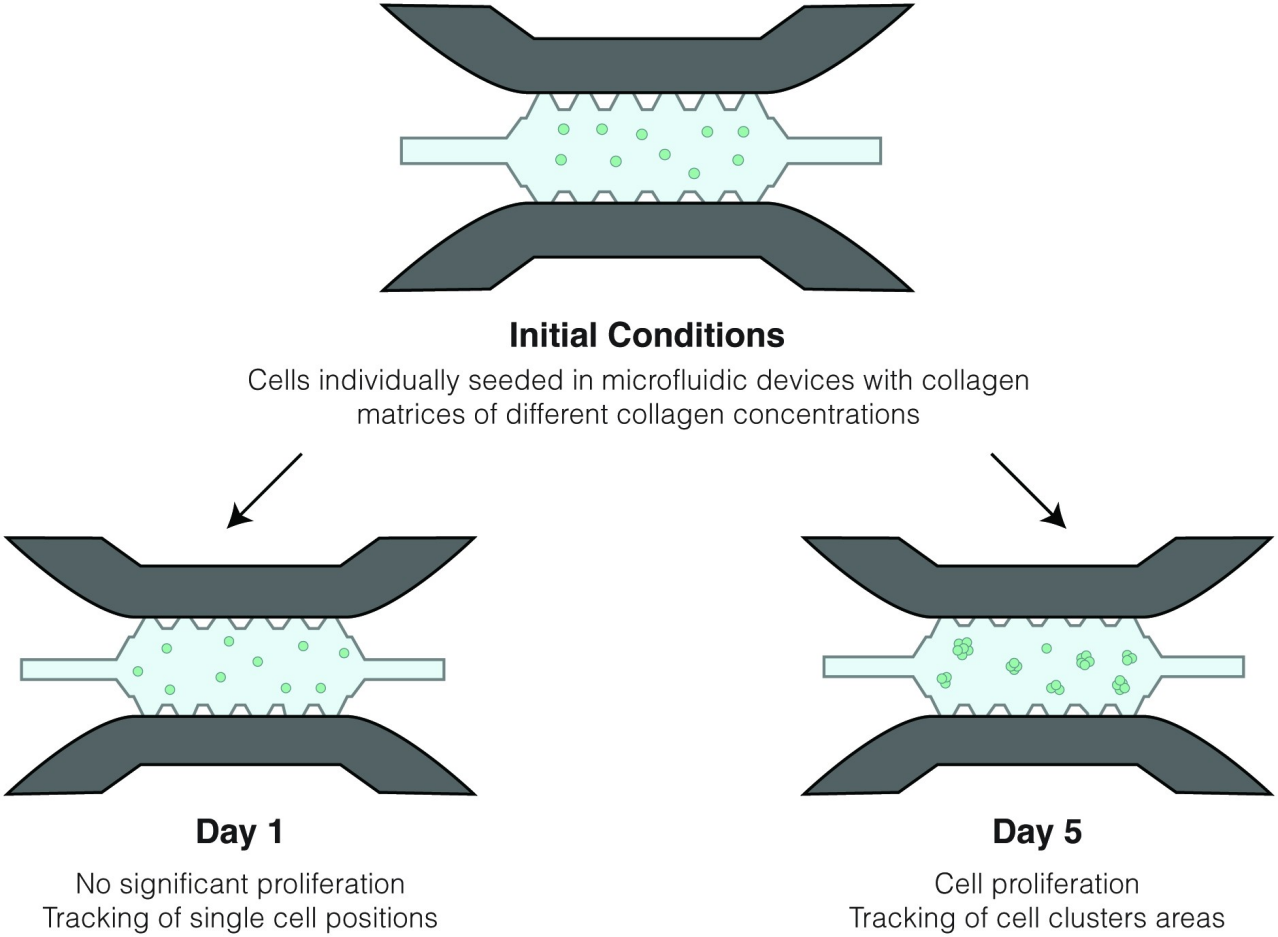
Schematic representation of the experimental configurations. Using the same experimental conditions, consisting of a microfluidic device in which cells were seeded in a collagen matrix, it was possible to characterize cell motility and cluster formation as two distinct processes. The results recorded at day 1 provided the data used to define locomotive forces and, consequently, cell-matrix interactions. Furthermore, the results from the 5th day of experiments were used to calibrate cell-cell interactions.

Regarding the formation of multicellular structures, the authors followed the same experimental procedures as mentioned above but allowed for cells to proliferate for seven days. In this case, the cell motility metrics were not tracked, and the focus shifted to quantifying how the tumour spheroid size evolved over time. Thus, cluster areas were tracked at time points of 1, 3, 5 and 7 days, and the obtained results were compared between the different matrix density values. The authors also analysed how the cluster eccentricity, measured at day 7, may vary according to the microenvironment. A schematic representation of the experimental conditions is presented in Fig 1.

This experimental study resulted in two key conclusions. First, in regard to single-cell migration, the results showed that the motility was hindered in matrices with high collagen concentrations. In fact, as the collagen density increased, cells encountered more difficulties moving through the ECM due to steric hindrance. Accordingly, cells became increasingly more confined, leading to shorter travel distances and lower cell velocity values. Second, the results showed that the collagen concentration also regulates tumour formation and growth, mainly through its effect on individual cell migration. On the one hand, cells seeded in low-density matrices disseminated through the matrix, forming small clusters. On the other hand, when cell motility became hindered due to high collagen concentrations, large multicellular structures started developing.

Our goal was to introduce the general trends and results on this topic published in [39] through a tumour growth computational model. Namely, we aimed to develop a model that enables users to define different collagen concentrations, analyse how matrix density restrains motility and understand how this situation favours tumour growth. Accordingly, we based the design of our model on reproducing the same setups as those seen experimentally and used the published data to calibrate our model. Although the results of previous studies are at the core of our model, we sought to build a flexible framework that can be extended to other models and scenarios.

### Computer-based modelling framework

The first step of the implementation process consisted of choosing an adequate modelling framework in concordance with our main objective. Since we considered that mechanical interactions should be regarded at the cellular level, a discrete model probably suits the problem better than a continuum approach would. However, even among discrete frameworks, there are multiple possible modelling choices, such as centre-based and deformable cell models [49]. The first assumes that cells are represented by their centre point, without accurately accounting for cell geometry, while the latter can describe the cell shape at the cost of higher complexity, which limits the number of cells that can be present for each simulation [52].

Discrete models are known to have an increased level of complexity, usually associated with higher computational costs [53]. Hence, researchers have developed hybrid continuum-discrete models to reduce computational costs while still accounting for cell-level information. Recently, PhysiCell [51] was presented as an open-source hybrid 3D cell simulator that models the cellular environment through a combination of a continuum approach, which describes the chemical microenvironment and the mechanical behaviour of the matrix, and an agent-based model that mimics the cells. For the continuum part of the model, an associated piece of software, BioFVM [54], is used to solve reaction-diffusion equations, which, for several user-defined substances, account for substance diffusion and decay, as well as the existence of both bulk and cell-centred sources and sinks.

Based on PhysiCell’s advanced state of development, along with its flexibility to include new user-defined modules, we have chosen to develop our model as an extension to this framework. To do so, we relied on built-in functions that account for tracking the cell volume, cell proliferation and cell death, and we extended the features that model cell motility and cell mechanics. Moreover, we introduced the ECM, which the PhysiCell system did not include. Thus, collagen was incorporated into the model as a non-diffusing substance that is defined by its density. Subsequently, this value was used to compute cell-matrix interactions, as further explained below. For the first approach, we assumed that the collagen density is uniform throughout the domain and that cells do not remodel the matrix, so the collagen concentration does not change over time. More details on the parameters used to implement the model are presented in Table 1.

**Table 1:** Reference parameter values for the model.

Symbol	Parameter	Value	Unit	References
$\rho_{O_{2}}^{0}$	Oxygen Initial Density	38.0	mmHg	[51, 54]
$D_{O_{2}}$	Oxygen Diffusion Coefficient	1.0e5	*μ*m^2^/min	[51, 54]
$\lambda_{O_{2}}$	Oxygen Decay Rate	0.1	1/min	[51, 54]
$U_{k,O_{2}}$	Oxygen Uptake Rate (per cell, *k*)	10.0	1/min	[51, 54]
$\rho_{Collagen}^{0}$	Collagen Initial Concentration	[2.5, 4.0, 6.0]	mg/mL	[39]
*μ*	Drag Coefficient	[7.96, 18.42, 39.15]	Pa ⋅ s	[55]
*R*	Cell Radius	8.4	*μ*m	[51]
*R*_*A*_	Maximum Cell Adhesion Distance	1.25*R*	*μ*m	[51]
*c*_*cca*_	Cell-Cell Adhesion Coefficient	7.2	-	[51], estimated
*c*_*ccr*_	Cell-Cell Repulsion Coefficient	380	-	[51], estimated
*T*_*Ki*67−_	Cell Quiescence Time	6.5	h	[51], estimated
*T*_*Ki*67+_	Cell Proliferation Time	15.5	h	[51], estimated
*r*_*D*_	Cell Death Rate	0.00319	1/h	[51]

In addition, we modelled oxygen as a diffusing substance that degrades over time and that is consumed by cells. Changes in the oxygen concentration, $\rho_{O_{2}}$, were modelled in accordance with a reaction-diffusion equation:

$$\begin{array}{c} \frac{\delta \rho_{O_{2}}}{\delta t}=D_{O_{2}}\nabla^{2}\rho_{O_{2}}-\lambda_{O_{2}}\rho_{O_{2}}-\sum_{cells,k}\delta \left(x-x_{k}\right)W_{k}\left(U_{k,O_{2}}\rho_{O_{2}}\right)\;\text{in}\;\Omega \end{array}$$ (1)

with Dirichlet boundary conditions in *δ* **Ω**. Here, $D_{O_{2}}$ is the oxygen diffusion coefficient and $\lambda_{O_{2}}$ is its decay rate. In addition, for a cell *k*, δ(x) is the Dirac function, $x_{k}$ is the cell position, *W*_*k*_ represents the cell volume and $U_{k,O_{2}}$ is the cell consumption rate for oxygen. The parameter values for this equation can be found in Table 1. Furthermore, we assume that cell volume (*W*_*k*_) changes through time and is defined by PhysiCell’s custom volume functions, which are used as fully described in the original publication [51], and that allow the model to capture the changes in cell volume upon cell duplication and cell death. In particular, cell volume is assumed to start doubling once the cell enters the cell cycle, and it is halved when cells divide. Furthermore, its value decreases until reaching zero when cells enter apoptosis.

Additionally, through the use of built-in functions of the PhysiCell framework, we considered that oxygen shortage could lead to changes in the cells’ phenotype. In particular, we assumed that low oxygen levels (up to 15 mmHg) can induce a decrease in cell proliferation rates and promote cell death through necrosis. Since the experimental settings correspond to a well-oxygenated environment, assuming conventional cell culture oxygen values as well as the high permeability of PDMS-based microfluidic chips [56], and given that the cell concentration is not high, we consider that oxygen’s effect on spheroid formation can be disregarded. Nonetheless, an extended study of the oxygen levels in our simulations is provided in S1 Appendix.

**Fig 2:**
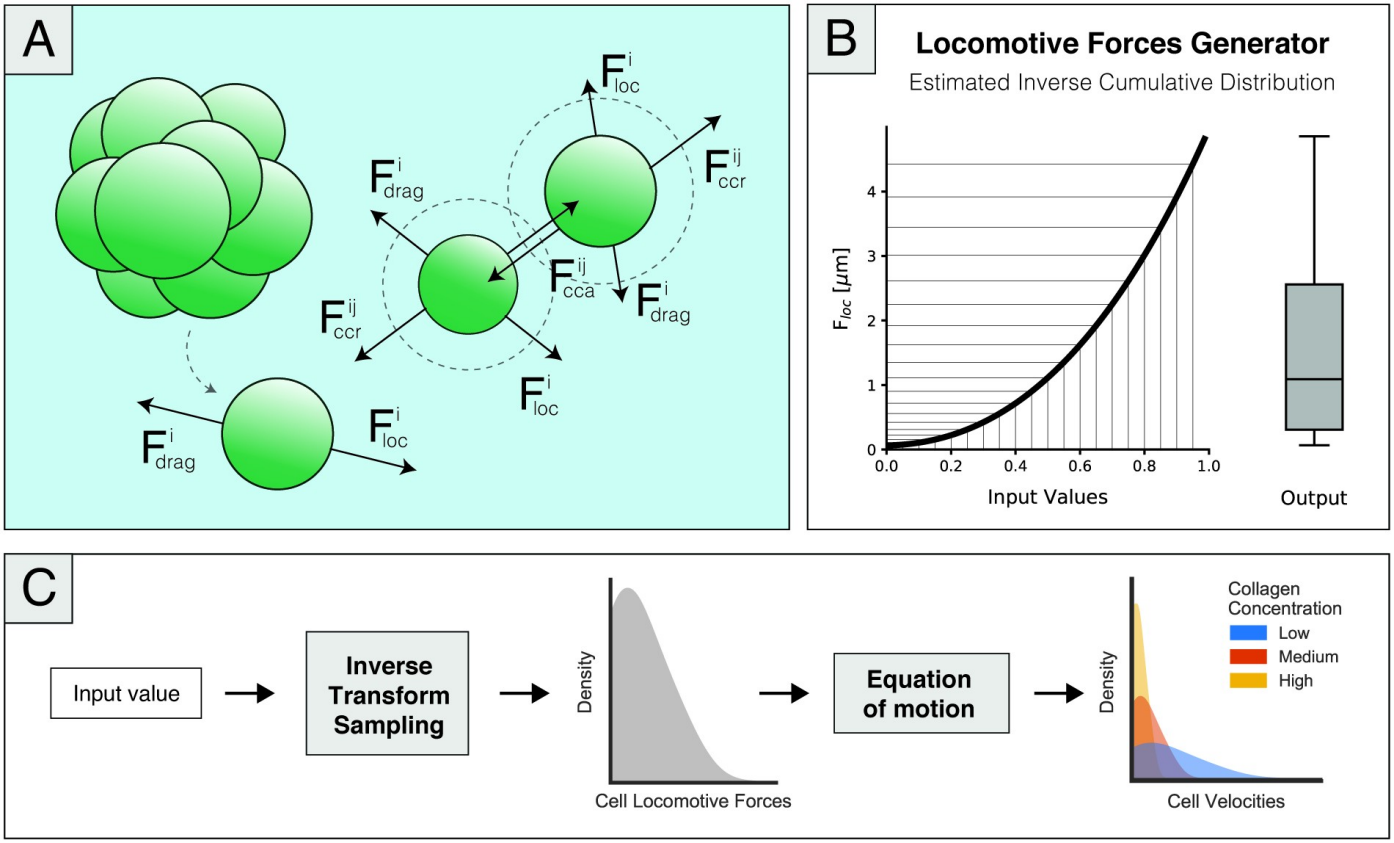
(A)A simplified representation of the force diagram showing the cell-cell and cell-matrix interactions present in the model, as described by Eq 2. The model considers cell-generated locomotive forces, drag forces imposed by the ECM, and cell-cell adhesion and repulsion of cells that are inside an interaction radius. Although the model only accounts for the cell volume and not cell geometry, spherical geometry is assumed. (B) Representation of the locomotive forces generator function modelled as 5 through an estimation of the inverse cumulative distribution of experimental cell velocities. When fed uniformly distributed random values between 0 and 1, the represented function produced a new set of values that followed the desired force distribution, as shown by the representative boxplot showcased as the output of this function. (C) Schematic representation of the implemented workflow to model cell-generated locomotive forces (considering no cell-cell interactions). At an average of 20 simulated minutes, cells are allowed to change their velocity both in magnitude and direction. Accordingly, at those time points, we generated a new cell-generated force value through the inverse sampling method. The output of this function was subsequently incorporated into the equation of motion, given by Eq 4, producing three different velocity distributions, each corresponding to a matrix density value.

Regarding cell-matrix mechanical interactions, the cell positions were updated by computing the effect of cell-cell forces acting on each cell, as represented in Fig 2. Accordingly, considering a cell *i* interacting with some neighbouring cells, N(i), we can consider the equilibrium equation taking the over-damped assumption:

$$\begin{array}{c} 0\approx \sum_{j\in N(i)}\left(F_{cca}^{ij}+F_{ccr}^{ij}\right)+F_{drag}^{i}+F_{loc}^{i} \end{array}$$ (2)

where $F_{cca}^{ij}$ and $F_{ccr}^{ij}$ are cell-cell adhesive and repulsive forces, $F_{drag}^{i}$ represents dissipative forces (such as fluid drag and matrix adhesion) and $F_{loc}^{i}$ is the net locomotive force. We assume that cell-matrix forces can be described through Eq 3:

$$\begin{array}{c} F_{drag}^{i}=-\mu v_{i} \end{array}$$ (3)

where **v**_*i*_ is the cell velocity and *μ* represents the drag coefficient. The mechanical properties of the collagen matrices used in [39] have been characterized in a previous publication [55] through rheological analysis. In this particular study, the authors characterized the viscoelastic properties of collagen matrices using a stress-controlled rheometer and showed that the dynamic viscosity, which measures the material’s resistance to flow, increases with collagen density. Hence, we expect cells to undergo increased difficulty when migrating in denser, and consequently more viscous, matrices.

Taking this expectation into account, we consider that the drag coefficient, *μ*, can be estimated using the data provided in [55]. Specifically, for each collagen density, we defined *μ* as the mean value for the measured dynamic viscosity values, as presented in Table 1. The viscosity values were considered to be uniform throughout the domain since we assumed that, as a first approach, the collagen density was also uniform.

Accordingly, the equilibrium equation was simplified to Eq 4.

$$\begin{array}{c} v_{i}=\frac{1}{\mu }(\sum_{j\in N(i)}\left(F_{cca}^{ij}+F_{ccr}^{ij}\right)+F_{loc}^{i}) \end{array}$$ (4)

which indicates that apart from the effect introduced by the ECM (which here is assumed to be a constant value), the cell velocity is only dependent on cell-cell interactions as well as the locomotive forces generated by the cell, both of which we need to characterize.

[39] presents two experimental configurations that refer to single-cell and multicellular studies. In regard to the individual cell motility studies, it is stated that cell-cell forces do not have a significant effect on cell trajectories, as the cells have enough space to migrate without interacting with each other. Taking this idea into consideration, we assumed **F**_*cca*_ and **F**_*ccr*_ in Eq 3 to be zero and considered that cell-generated and drag forces are dominant in this configuration. As mentioned previously, we considered drag forces to be represented by a constant value for each of the collagen concentrations. Therefore, we stipulated that by replicating this experimental setup (Setup 1), we could use the corresponding results to characterize the cell-generated locomotive forces.

Additionally, [39] introduces a second experimental setup that builds upon the previously described configuration. In this case, cells were allowed to proliferate for seven days, which enabled the development of relevant cell-cell interactions. Nonetheless, we postulate that cell-cell interactions do not significantly affect cell-generated forces. Instead, we believe that the cell motility in this configuration is a result of the cell-generated forces described by the previous setup, drag forces and cell-cell interactions. Accordingly, we replicated the new experimental conditions and calibrated cell-cell interactions to predict tumour growth.

We discuss both setups in more detail in the following subsections, and a summarized overview is presented in Fig 2.

### Defining cell-generated locomotive forces

The first set of simulations aimed to define how single cells migrate in 3D space and to assess whether the model can capture the effect that the collagen concentration has on migration. Experimentally, cell trajectories and cell speeds were tracked for a period of 24 hours, with the results showing that the collagen density reduces cell motility. We considered a single cell for each simulation, taking into account that we do not consider cell-cell interactions to affect cell motility. We tracked this cell’s trajectory and recorded spatial data at the same time points as those considered in the experiments (i.e., every 20 minutes).

To meet the requirements of this study, namely, avoiding cell-cell interactions, we consider that cells cannot duplicate. Moreover, given that the expected lifetime of the cells surpasses these 24 hours, cells were programmed not to enter cell death. This simulation setting was used to run 80 replicates for each of the matrix density values, resulting in a similar number of data points to those obtained experimentally.

**Fig 3:**
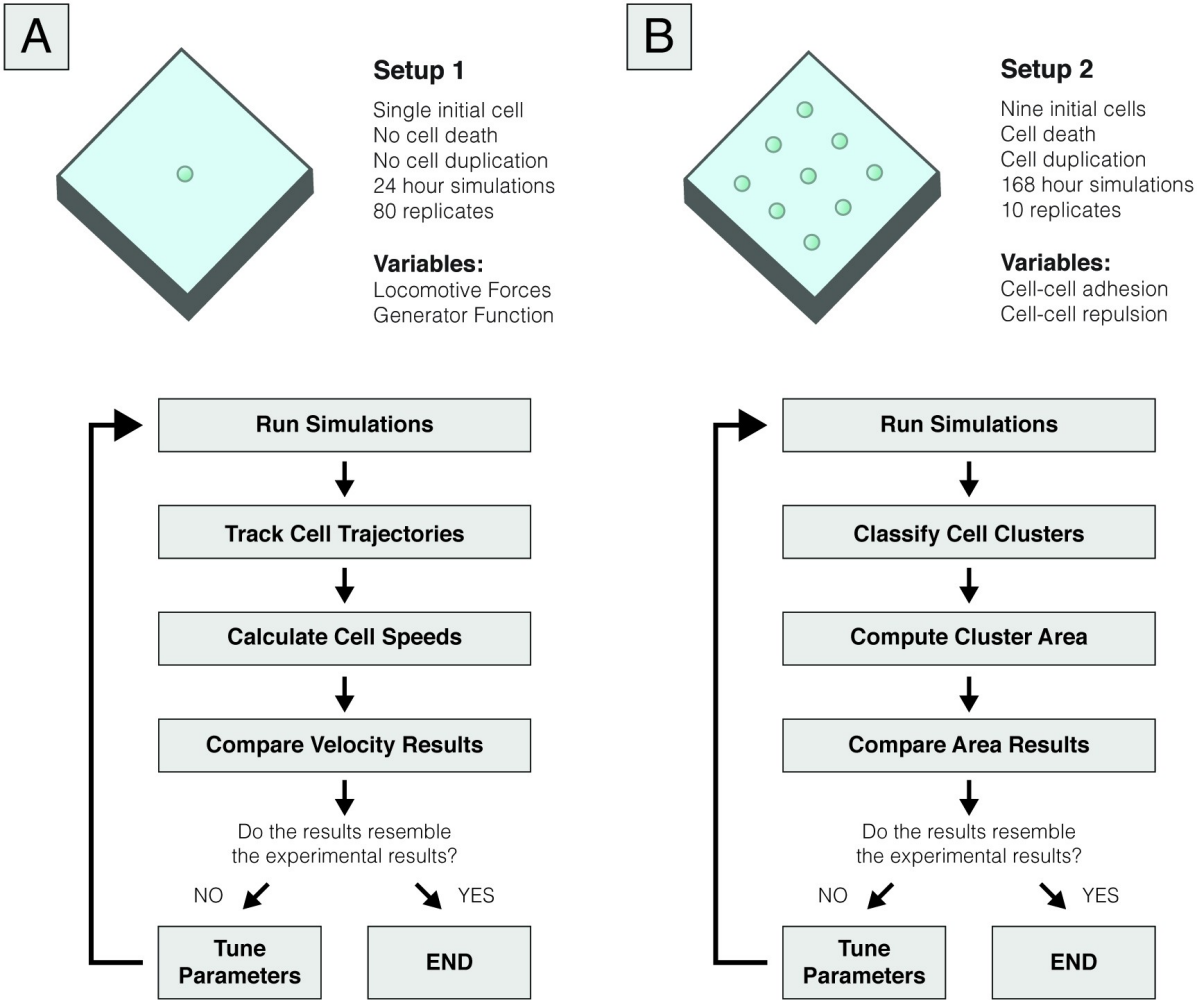
Schematic representation of the simulated conditions. (A) Representation of the single-cell migration simulation setup, which consisted of tracking the positions of an individual cell during 24 hours to calibrate cell-generated locomotive forces. Due to the short duration of the simulations, cell death and proliferation were both neglected. We used spatial data to compute the cell’s average and effective velocities, which were compared to the experimental data. Based on this analysis, we iteratively changed the parameters of the locomotive force generator function, given by Eq 5, until the results were consistent with the experimental data. (B) Representation of the second simulated setup, which aimed to define cell-cell interactions by assessing the development of multicellular clusters for seven days. Unlike Setup 1, cell death and proliferation were considered in this setup. We computed cluster area values at days 1, 3 and 5 of simulation by classifying the cells through a clustering algorithm and compared these data to the experimental results. Similar to what we did in Setup 1, we iteratively ran simulations to approximate our model to the experimental data, this time focusing on the parameters that modulate cell-cell interactions, namely, cell-cell adhesion and cell-cell repulsion.

Regarding cell-generated locomotive forces, the results in [39] show that there is a diversity of behaviours in cell motility, as seen by the non-uniform distribution of cell velocities. Using the simple setting previously described, Eq 4 shows that the velocity at which cells move depends solely on the viscosity of the collagen matrix, *μ*, and the locomotive forces generated by the cells. Given that we assume that this viscosity is uniform for each collagen concentration and given by a constant *μ*, it becomes apparent that the heterogeneity in behaviour should come from the locomotive forces. Thus, we defined that the distribution of forces should resemble the distribution of velocities, which was experimentally characterized. A schematic representation of this simulation setup and a summarized overview of our workflow to define cell-generated forces is present in Fig 3A.

Accordingly, we developed the model so that, at an average of 20 min, cells may change their motility, both in direction and in magnitude. Regarding the latter change, we needed to create a function that generated random values following the force distribution that, in turn, would produce the velocity distributions measured in the experiments. We chose to solve this problem through the inverse transform sampling method, which is generally used to obtain random values from any probability distribution. Through this method, the inverse cumulative distribution is computed, and by feeding this function uniformly random numbers between 0 and 1, it will output a range of values that follows the distribution that we aim to model. Fig 2B presents a representation of how the inverse cumulative function is used to obtain a value distribution of choice by taking a range of values *x*, which follow a uniform distribution between 0 and 1.

Henceforth, we created a force generator function by estimating an inverse cumulative density function from the experimental cumulative density distribution, which ultimately resulted in Eq 5:

$$\begin{array}{c} F_{loc}\left(x\right)=1.56x^{3}+3.27x^{2}+0.07x+0.06 \end{array}$$ (5)

It must be noted that, as stated previously, our objective was to capture the general tendencies seen experimentally and not to single-handily replicate the experimental data on a small scale. Hence, we chose to estimate a general curve based on the three available empirical cumulative cell velocity distributions presented in [39] (for the different collagen concentrations), so that it could provide good results for all collagen densities, rather than fit the model to each curve. Subsequently, we iteratively changed its coefficients based on the obtained velocity distributions, so that the results resembled those seen experimentally. An extensive study on how these parameters may influence our results can be found in S2 Appendix. Furthermore, this analysis also includes an overview of how the model may respond to other density distribution functions.

Finally, to compute the direction of these forces, it was assumed that the cells adopted a completely random walk. Therefore, every time the velocity of a cell *i* was updated, a unit vector that defines a random direction, **e**^*i*^, was computed. Afterwards, this value was multiplied by the magnitude value given by Eq 5, thus defining $F_{loc}^{i}$:

$$\begin{array}{c} F_{loc}^{i}=F_{loc}e^{i} \end{array}$$ (6)

Similar to what was done experimentally, we computed the mean cell velocity by taking the average value of cell velocities, calculated as the distance travelled between time points, divided by the recording time. Additionally, we computed effective cell velocities as the distance between the initial and final positions of the cell, divided by the time of the simulation.

### Defining cell-cell interactions

Cell-cell interactions are characterized by potential functions as in [51] and are regulated by adhesion and repulsion coefficients, *C*_*cca*_ and *C*_*ccr*_, respectively (see Table 1). In particular, adhesion and repulsion forces between two cells *i* and *j*, separated by a distance of (x_*i*_ − x_*j*_), represented as **r**, are given by Eqs 7 and 8:

$$\begin{array}{c} F_{cca}^{ij}=C_{cca}\nabla \phi \left(r\right) \end{array}$$ (7)

$$\begin{array}{c} F_{ccr}^{ij}=C_{ccr}\nabla \psi \left(r\right) \end{array}$$ (8)

for which ∇*ϕ* and ∇*ψ*, respectively, can be written as:

$$\begin{array}{c} \nabla \phi \left(r\right)=\{\begin{array}{cc} {\left(1-\frac{\left|r\right|}{R_{A}}\right)}^{2}\frac{r}{\left|r\right|} & \text{if}\;|r|\leq R_{A}\\ 0, & \text{otherwise} \end{array} \end{array}$$ (9)

$$\begin{array}{c} \nabla \psi \left(r\right)=\{\begin{array}{cc} -{\left(1-\frac{\left|r\right|}{R}\right)}^{2}\frac{r}{\left|r\right|} & \text{if}\;|r|\leq R\\ 0, & \text{otherwise} \end{array} \end{array}$$ (10)

where *R*_*A*_ represents the maximum distance between two cells at which adhesion forces are present, and *R* is the maximum distance for which repulsion forces are present. *R* is, simultaneously, the radius of a single cell. Below these values, interaction forces between cells are assumed to be null.

Hence, as presented in Fig 3B, we aimed to calibrate these cell-cell interaction parameters by analysing the formation of multicellular clusters, and their subsequent growth, as depicted by the experimental quantification of cell area values. Accordingly, we placed nine initial cells in the domain at a distance that would enable individual cluster growth, without promoting the junction of two different clusters, as this was not observed experimentally. Unlike the previously described setup, cells were allowed to proliferate and undergo apoptosis. Specifically, it was considered that cells could either be in a quiescent or a proliferative state, which would correspond to Ki67- and Ki67+, respectively [57]. Taking into account the cell state for a cell *i*, represented as $S_{i}$, the probability of the cell in a Ki67- phase entering a Ki67+ phase in a time interval [*t*, *t* + Δ*t*] can be given by:

$$\begin{array}{c} \text{Prob}\left(S_{i}\left(t+\Delta t\right)=\text{Ki}67+|S_{i}\left(t\right)=\text{Ki}67-\right)=1-e^{-\frac{1}{T_{\text{Ki}67-}}\Delta t}\approx \frac{\Delta t}{T_{\text{Ki}67-}} \end{array}$$ (11)

where *T*_ki67-_ represents the time spent in the Ki67- state. Similarly, the probability of a proliferative cell to enter a quiescent state is:

$$\begin{array}{c} \text{Prob}\left(S_{i}\left(t+\Delta t\right)=\text{Ki}67-|S_{i}\left(t\right)=\text{Ki}67+\right)=1-e^{-\frac{1}{T_{\text{Ki}67+}}\Delta t}\approx \frac{\Delta t}{T_{\text{Ki}67+}} \end{array}$$ (12)

Regarding cell death for both states, the probability of a cell entering apoptosis, represented by a state D, is given by the death rate, *r*_*D*_, as described in Eq 13:

$$\begin{array}{c} \text{Prob}\left(S_{i}\left(t+\Delta t\right)=D|S_{i}\left(t\right)=\text{Ki}67-/+\right)=1-e^{-r_{D}\Delta t}\approx r_{D}\Delta t \end{array}$$ (13)

The time steps (Δ*t*) used in our simulations are coherent to those originally used in the PhysiCell framework [51]. Accordingly, the time step used for cell processes such as described above, namely cell death and cell cycle, is in the order of minutes (6 min), whereas the time steps used for the diffusion and mechanical analyses are, respectively, 0.01 and 0.1 min.

For this setup, simulations were run for seven simulated days and recorded at time points of 24 hours, with a total of 10 replicates for each matrix density value. At time points of 24, 72 and 120 hours, cells were classified into clusters. Experimentally, the results were obtained through a manual classification procedure, using an image-based piece of software. Several microscopy images were taken, representing different slices of the microchip height. Subsequently, the subregion that better captured the area of the clusters was chosen and used as input for a classification algorithm. The authors selected the multicellular clusters’ approximate locations in the images of interest, and the algorithm identified the regions of interest and computed the cluster area by fitting a circle to the detected structure.

However, we adapted this procedure to our data and decided to automate this process since we needed to iteratively run simulations and obtain cluster metrics to assess how cell-cell interactions could be calibrated to obtain better results. To obtain comparable data representations to those presented in [39], we used the x and y coordinates of cells present in a height-defined z-region. We only used a section of the z-axis to obtain a similar selection of cells to what would be expected from a confocal microscopy analysis, disregarding cells that would be out of focus. With this spatial data, we used an implementation of the density-based spatial clustering of applications with noise (DBSCAN) algorithm [58] to classify each cell into a cluster or identify it as an outlier. In particular, we used the implementation offered by scikit-learn [59], a publicly available Python module for machine learning algorithms. DBSCAN is a distance-based algorithm that requires users to define the minimum number of cells in a spheroid and a radius of interest. Consequently, we inferred these values, which are presented in Table 2, from experimental observations. An extended sensitivity analysis of the effect of these parameters on our results can be found in S2 Appendix.

**Table 2:** Parameter values for the post-processing of results.

Parameter	Values	Unit	References
z-Height of Interest	[-48—48]	*μ*m	Estimated
DBSCAN Radius	18	*μ*m	Estimated
DBSCAN Minimum Number of Cells	3 (2 at day one)	-	Estimated

Having defined the clusters, which were visually assessed to avoid misclassifications, the area of each cluster was computed. Here, we aimed to replicate the procedure used in [39], in which the cluster area is computed by calculating the area of the circle that best fits each cluster. Accordingly, we started our analysis by calculating the centroid for each cluster, which we defined by the average x and y coordinates of all the cells that formed the cluster. Subsequently, we computed the distance of each cell to that point, which we used to estimate the radius of the cluster by calculating their average value. Using the estimated cluster radius, we computed the area for a circular geometry.

Last, after seven simulated days, we also computed cluster eccentricity by identifying an equivalent ellipse with the same second-moments as the region defined by the cell centres of each cluster. We calculated ellipse eccentricity as the ratio of the distance between the foci of the ellipse and its major axis length, which we assumed to be representative of the cluster eccentricity. In contrast to our previous strategy, we analysed the entire height of the domain, as we observed that at this point of development, the geometry of the clusters did not significantly change based on the selected subsection. Since the experimental eccentricity values considered only spheroids with an area larger than 1000 m^2^ and were obtained through a semi-automatic classification algorithm, the computational results were also manually evaluated to disregard clusters with small area values or that were misclassified by the automatic algorithm.

We remark that, in order to extend the model and replicate both setups, we have relied on PhysiCell’s ability to scale and efficiently compute the dynamics of multiple substances and cells. Likewise, although we built the additional functions to be in line with the standard PhysiCell features, we did not focus on reducing computational times and costs. However, it must be stated that we have not experienced a substantial increase in computational times as a result of our numerical implementations.

## Results

### Increasing values of ECM density hinder individual cell migration

As previously stated, this set of simulations aimed to define cell-generated forces and to evaluate the model’s ability to qualitatively describe the effect of matrix density on individual cell migration. Regarding the locomotive force distribution, our modelling choice was heavily based upon how the experimental observations showed that velocity values exhibited a positively skewed distribution, a trend that is in concordance with previous studies, [11, 60]. In a biological sense, this result indicates that cells are more likely to migrate at lower speeds. However, in a few instances, cells may migrate at much higher velocities than the general trend seen for that set of conditions. This result could also be indicative of a low motile fraction, but this is currently not implemented in the model; i.e., all cells are programmed to migrate with similar behaviour.

**Fig 4:**
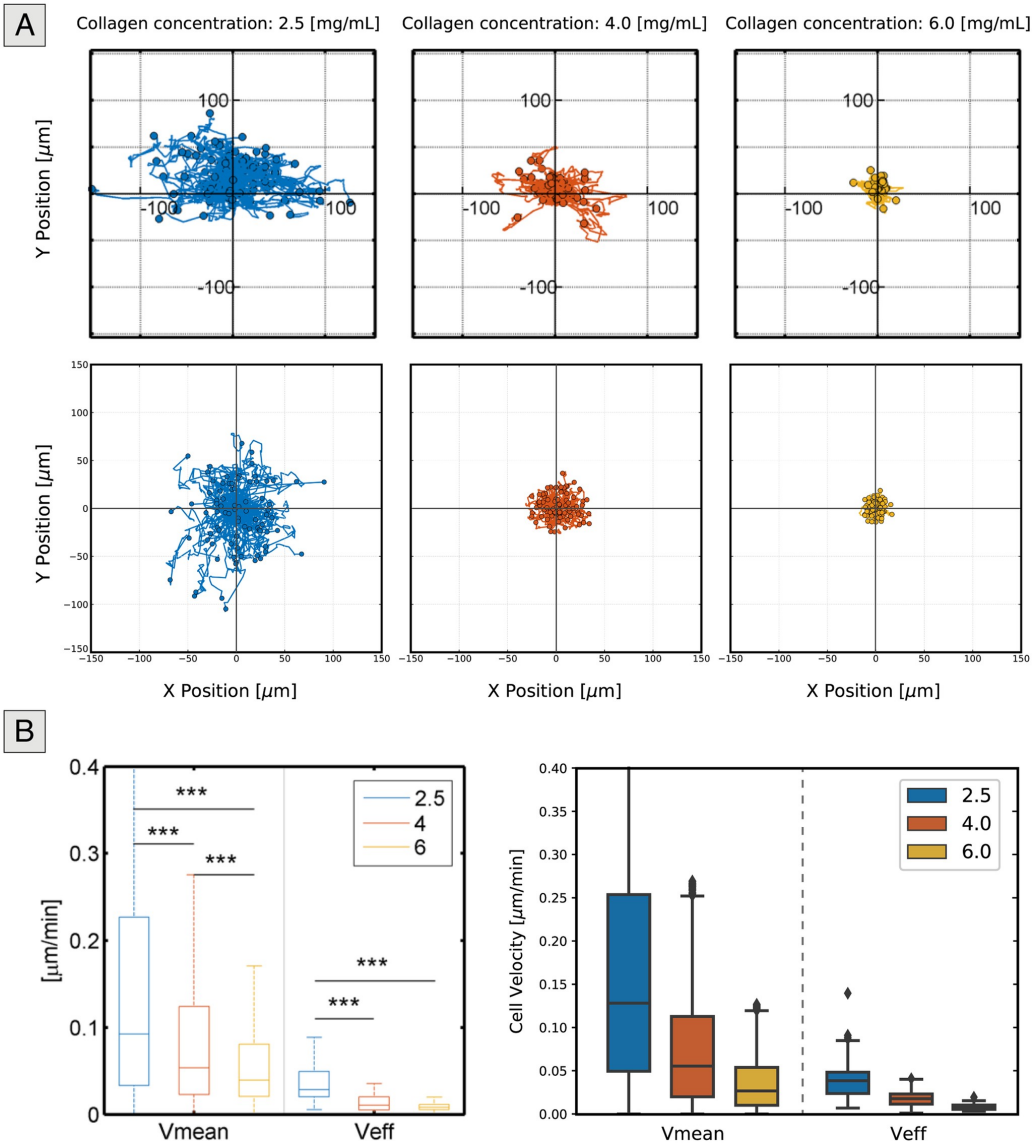
Experimental and simulated results for the individual migration setup. (A) Representation of relative cell trajectories for the experimental (top) and computational (bottom) results. As the density of the collagen matrix increases, cells become more confined, resulting in reduced cell movement. (B) Mean and effective cell velocities for cells seeded in matrices of varying collagen density. Both the experimental (left) and computational (right) results indicate that as the density increases, cells travel shorter distances due to the restrictions imposed by the matrix and both the mean and effective speeds decrease.

Fig 4 shows that the model generally captures this non-uniform distribution of cell velocities quite well. As expected, the distribution does not apply to each of the density values specifically, since we used a general approximation, rather than a specific distribution for each matrix density value. However, we show that good results can be achieved without the need for an increased level of complexity. In addition, Fig 4 and Table 3 show that our simulation results replicate the effect of high ECM density values on individual cell migration.

**Table 3:** Statistical data of the mean and effective cell velocities for different collagen concentrations for both experimental settings and computational simulations.

**V_mean_ [*μ*m/min]**	**N**		**median**		**mean**		**se**	
**Concentration** [mg/mL]	Exp	Comp	Exp	Comp	Exp	Comp	Exp	Comp
2.5	5181	5840	0.0922	0.1281	0.1696	0.1702	0.0020	0.0019
4.0	3807	5840	0.0537	0.0553	0.1040	0.0746	0.0023	0.0009
6.0	3837	5840	0.0392	0.0266	0.0630	0.0355	0.0023	0.0004
**V_eff_ [*μ*m/min]**	**N**		**median**		**mean**		**se**	
**Concentration** [mg/mL]	Exp	Comp	Exp	Comp	Exp	Comp	Exp	Comp
2.5	82	80	0.0285	0.0390	0.0365	0.0421	0.0020	0.0026
4.0	57	80	0.0105	0.0182	0.0136	0.0185	0.0024	0.0010
6.0	60	80	0.0079	0.0082	0.0086	0.0086	0.0024	0.0004

It can be seen that the cell trajectories become more contained as the collagen concentration increases. Furthermore, the results show that both mean and effective cell velocities adopt smaller values as matrices present higher density values. It is important to state that regarding individual cell trajectories, the experimental results show that cells appear to spread out in a horizontal direction. We stipulate that this behaviour is promoted by the alignment of the collagen fibres, which is a result of the procedures used to fill out the microfluidic chips and collagen polymerization and has been shown to regulate cell migration by promoting more directed migration patterns [61]. In contrast, simulated cells adopt more random motility patterns. However, this outcome is expected since we did not define a preferential direction of migration, as our focus was studying cell movement in general and not replicating individual cell trajectories. Nonetheless, considering that it has been shown that fibre alignment can affect cell motility [61, 62], the model could be easily extended to consider directionality through Eq 6 by defining a preferential direction, rather than a random vector. In terms of distance travelled, the computational and simulated results are similar.

We note that although these results are presented through 2D plots, cells migrate in 3D space in both the simulated and experimental conditions. However, experimental quantification was previously conducted using 2D imaging techniques, through confocal microscopy, and these results were presented in the form of 2D plots in [39]. Accordingly, we followed the same data processing procedures to facilitate the comparison between the experimental and computational results. Thus, to better showcase the model’s ability to model 3D cell behaviour, we have included a 3D animation as supporting information (see S1 Video).

### Matrices with a lower collagen concentration produce smaller structures due to cells’ ability to migrate

**Fig 5:**
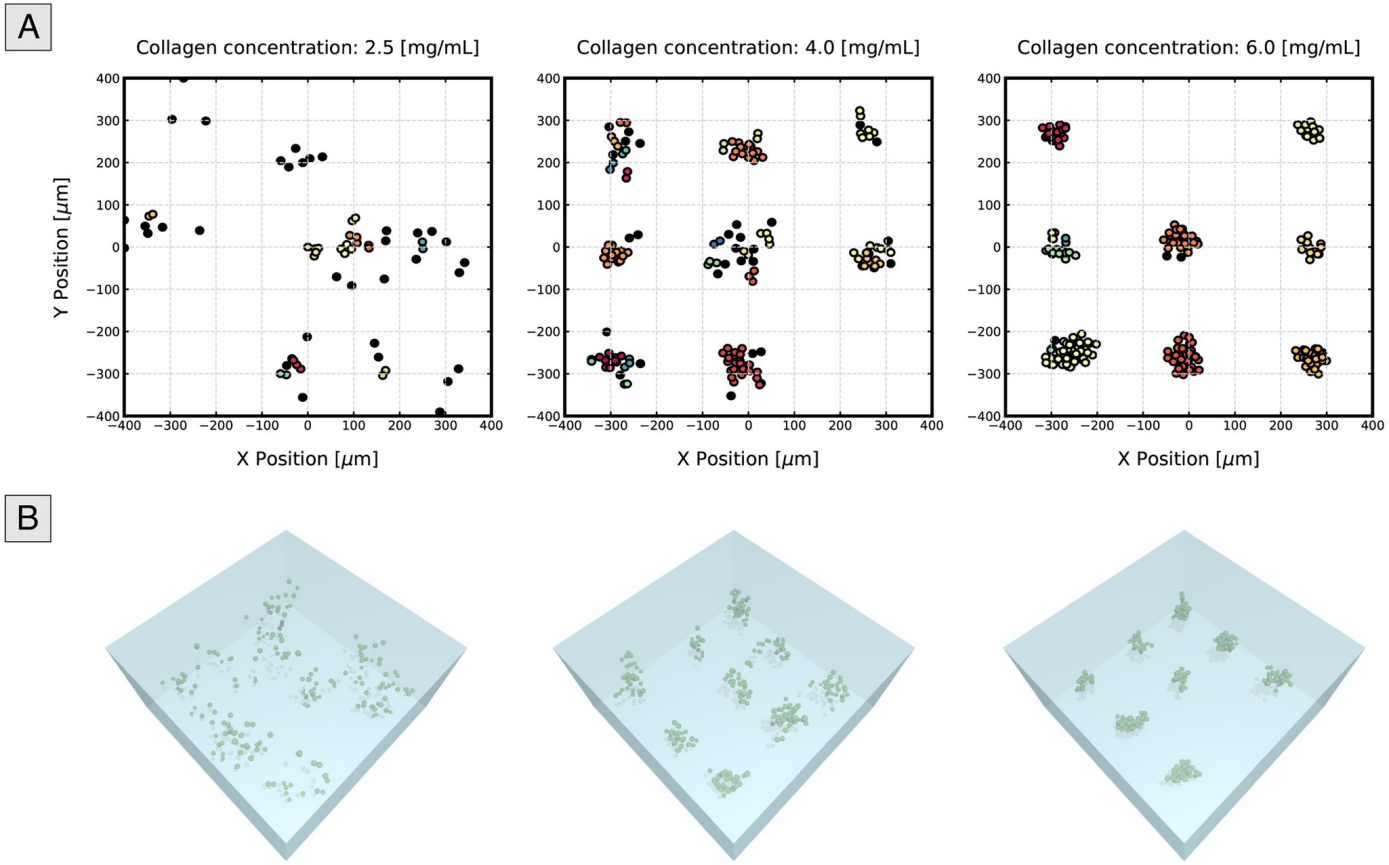
Representation of cell positions after five simulated days of tumour growth. 2D (top) and 3D (bottom) representations of the coordinates of cells grown in matrices of different collagen concentrations after five days. For the 2D scatter plots, we selected only cells present in a defined height of interest to remove cells that would otherwise be out of focus in microscopy images. A single replicate was chosen for each condition to produce these plots. Different colours represent different clusters, whereas black cells are those considered outliers (i.e., they do not belong to any of the cell groups). The cluster area increases with density, as cells stay closer to their original position. On the other hand, in low collagen density matrices (left), in which individual cell migration is not limited, individual cells are seen to stray away, resulting in a large number of outliers and smaller, sparser tumours.

Similar to the experimental conclusions, the results of our simulations describe how tumour size is affected by matrix density, as seen in Fig 5, which presents the cell positions in the 2D plane at the end of the fifth simulated day for each matrix density value. Coloured cells represent cells that belong to a cluster, with each colour corresponding to a different cell group, and black cells represent outliers. From the figure, it becomes apparent that cells seeded in matrices with high collagen density tend to be more confined and form clusters with higher area values than those grown at low densities, in which barely any spheroids are present.

Accordingly, an interesting dichotomy between migration and tumour size can be observed: for conditions where cell migration is allowed, the multicellular clusters experience less growth, and vice-versa. Previous studies have proposed a possible explanation for the interplay between migration and tumour growth through the “Go-or-Grow” mechanism, which may depend partially on the physical constraints of the ECM [63, 64]. According to this mechanism, migration and proliferation are temporally exclusive: migrating cells are not able to proliferate, resulting in clusters of a smaller size. Nonetheless, it is still unclear whether these two phenomena are not coincident in time [65]. Interestingly, our results suggest that there is no need for such a mechanism under the studied conditions. There is a preference for migration over tumour growth, in terms of tumour area, in matrices where the density allows for individual cell migration. However, we do not implement cell proliferation suppression in migrating cells. Henceforth, we highlight the difference between tumour growth and cell proliferation and recognize the effect of individual cell migration on the former, but not necessarily on the latter.

**Fig 6:**
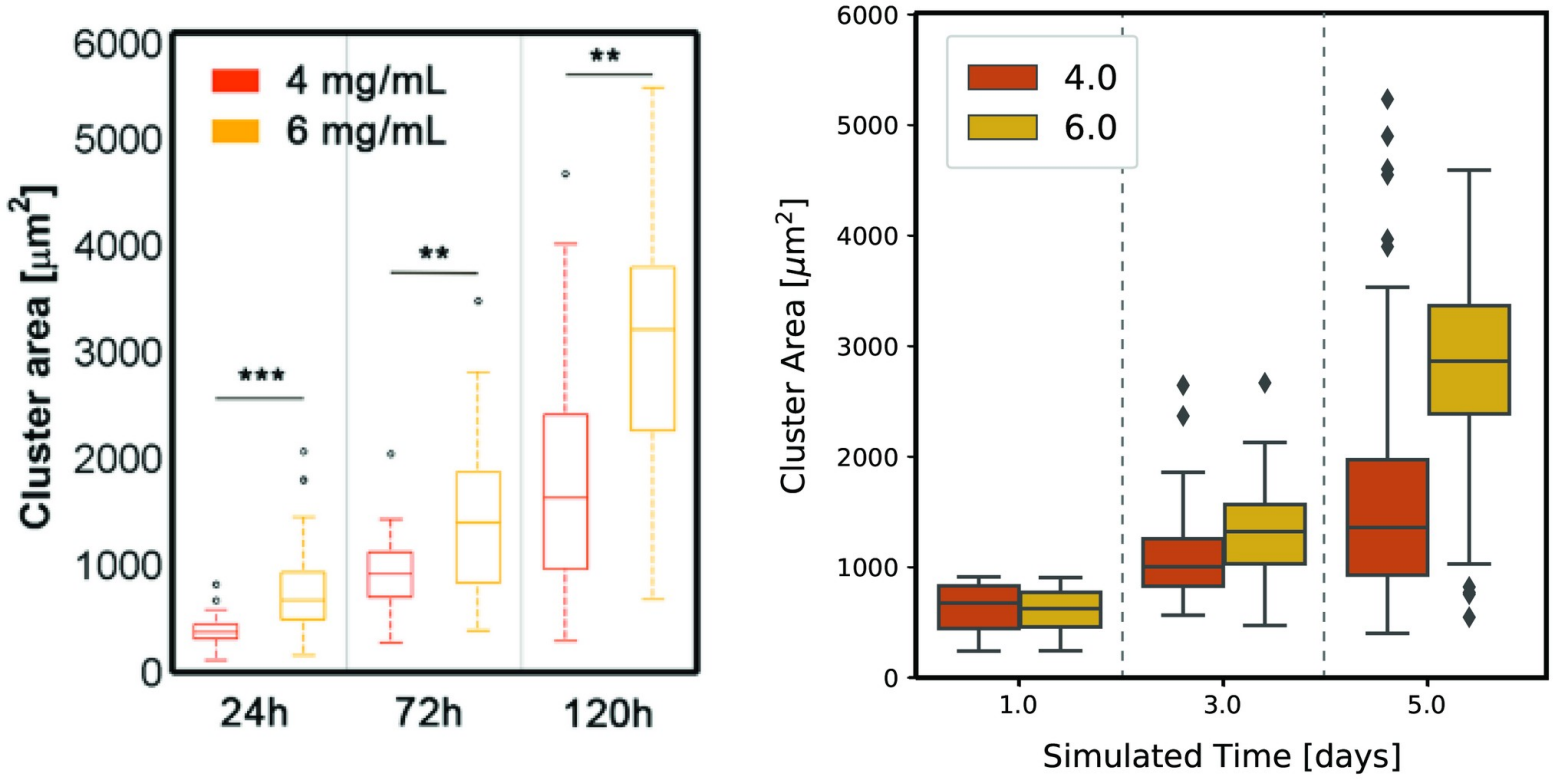
Evolution of cluster area growth over five simulated days. Distribution of cluster areas at the end of days one, three and five for clusters grown in matrices of medium and high collagen density (4.0 and 6.0 mg/mL, respectively), for the experiment (left) and computational (right) settings. These distributions take into account data from all five replicates. Cells seeded in collagen matrices of low density (2.5 mg/mL) did not show significant multicellular cluster formation and growth and hence were not represented. An increase in tumour size through time can be seen for both the medium and high collagen concentrations, but larger densities induce the formation of clusters of larger areas.

Fig 6 depicts the time evolution of cluster areas for matrices of medium and high density at days one, three and five and indicates that our model not only captures cluster area size after five days of growth but can also describe the evolution of tumour size through time. Only values for medium and high densities are shown since the number of clusters with a significant area in matrices of low density is not significant.

**Fig 7:**
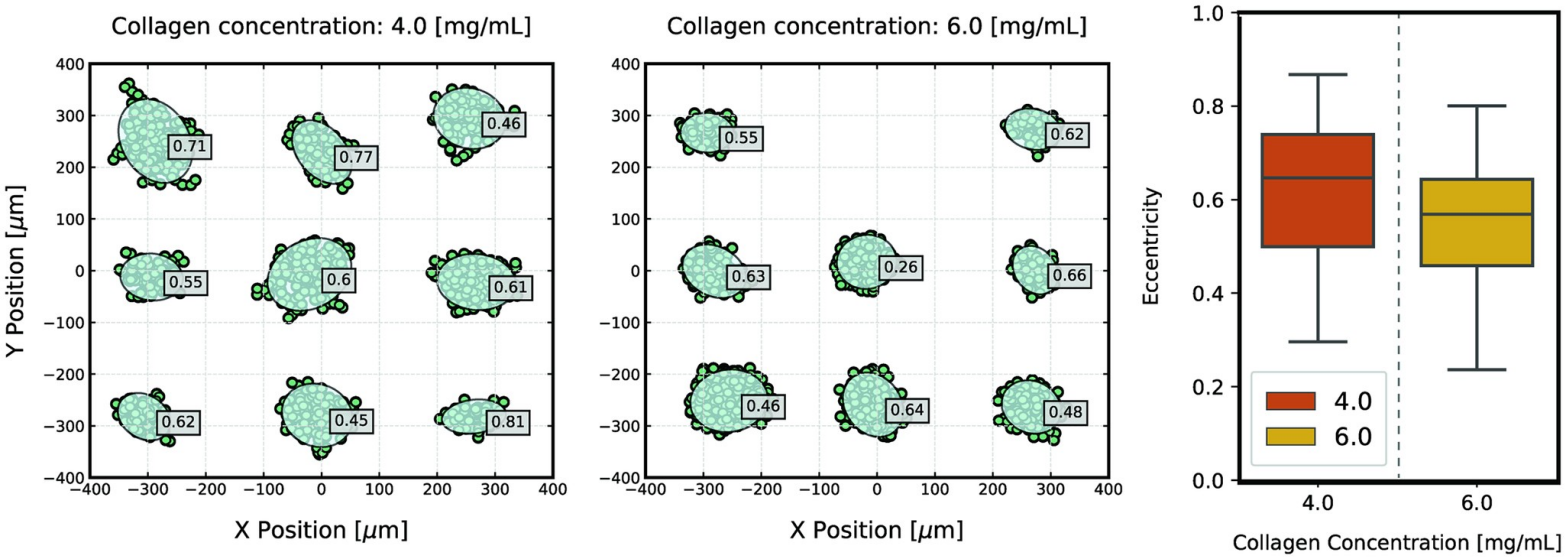
Cluster eccentricity after 7 days of growth. Representation of the xy cell coordinates for the cells present in the entire height of the domain, overlapped by the clusters’ equivalent ellipses and eccentricity values (left). Individual cells and clusters of small areas or areas that were manually evaluated as having been misclassified by the clusterization algorithm are not represented. Clusters of cells grown in collagen matrices of high density are shown to present smaller values of eccentricity (indicating a rounder morphology), while clusters grown in matrices with a lower collagen concentration adopt slightly larger values, as cells migrate away from the cluster, producing more elongated morphologies. The distribution of eccentricity values for both densities (right) further confirms this idea.

Finally, Fig 7 presents the results obtained for cluster eccentricity at seven days of simulation. As a general trend, the clusters adopt a relatively round morphology, which is further promoted in high-density matrices. Nonetheless, as cells become more able to migrate, the eccentricity values are seen to increase since cells start to stray from the original cluster but, ultimately, due to a decrease in cell speed, reconnect to the original structure as cells proliferate. Likewise, more elongated clusters start to appear, which may also be promoted by the alignment of collagen fibres. Nevertheless, the model does not capture this property of the collagen matrices, and the simulated cells adopt a random walk, which does not favor cluster elongation in a determined direction, but rather leads to tumour expansion in all directions. This is further promoted by the effect of cell-cell interactions, that may counteract individual cell movement as cells move in different directions. Consequently, the model is currently not able to predict a direct correlation between increased migration and strand-like morphology, which has been shown experimentally. A possible strategy to try to simulate the formation of elongated clusters would be to force directional motility instead of a random walk. Nevertheless, we think that, by itself, this is likely not sufficient for producing strand-like clusters, as all cells would migrate in this direction. Hence, the clusters would be expected to move collectively, and not to deform, which, in relative terms, would produce comparable results to those presented here. Consequently, we believe that a leader-follower mechanism [66, 67] has to be implemented so that only some cells migrate in a determined direction, pulling on neighbour cells to force the cluster to grow in that direction.

We note that the computational results for cluster eccentricity are, overall, relatively larger than those seen experimentally. Yet, we attribute this outcome to the intrinsic differences between the two datasets and the used algorithms for cluster classification, as we observe the same trends in each setup. It must be taken into account that the experimental measurements relied on semi-automatic imaging methods, which are pixel-based and, consequently, have lower sensitivity to perturbations in cell coordinates. These small differences can greatly influence the eccentricity values of clusters, especially when considering rounder structures, as we explain in more detail in S2 Appendix. Furthermore, the experimental methods focused more on the contours of the multicellular cluster, whereas we based our computational results on the coordinates of the cells’ centres, without taking into account cell geometry. Nonetheless, we consider that the model captures the dynamics of the experimental setup.

## Conclusion

In this study, we present a centre-based model extension to simulate individual and collective cell behaviour in which the regulatory effect of matrix density on cell migration and tumour formation is introduced, using previously published experimental data [39], to calibrate the model. Furthermore, we are able to qualitatively describe how an increase in matrix density leads to smaller cell velocity values and how this, in turn, suppresses the invasion of single cells from their original multicellular cluster, thus producing cell clusters of larger areas. In contrast, lower density values enable cell migration, resulting in sparser and smaller tumours.

We show that this relationship between cluster size and individual cell migration, often attributed to suppression in cell proliferation of migrating cells, can be achieved without changing the rates of cell proliferation [63, 64]. Accordingly, our results align with other studies that do not consider motility and proliferation to be mutually exclusive as well as with the idea that proliferative tumours can also be highly invasive [65].

We also introduce a representation of the distribution of cell-generated locomotive forces, which are fitted to resemble the experimental results in [39]. Using this model, we can capture the heterogeneity seen in cell behaviour in our data and other previously published experiments [11, 60], with cells generally tending to travel at low velocities, with a few also travelling at relatively high speeds. Since this model is fitted specifically to a set of experimental data related to a specific cell line and conditions, we consider that the model may not accurately describe experimental settings with different cell lines or different matrix density values. Nonetheless, we believe that the model could be easily updated by fitting parameters to other experimental results, while the main mechanisms should not need significant changes.

Regarding the limitations of our model, one of the shortcomings that we identified was its simplified depiction of collagen matrices. On the one hand, the model disregards the fibrous nature of collagen matrices and the effects that it may have on motion directionality, which was not studied here. On the other hand, we assume that the collagen matrices are homogeneous materials, which is not completely accurate for these experimental settings, in which there are local regions with higher and lower numbers of fibres [43, 55]. As a first approach, we are currently choosing to characterize drag forces through the value of the mean dynamic viscosity of the matrix for each collagen concentration. Nonetheless, we do know that viscosity values vary according to a normal distribution [55]. Accordingly, we could have defined the ECM not by constant viscosity values, but through this spatial distribution, which would provide us with a better description of matrix heterogeneity. However, instead, we considered the ECM as a substance (i.e., collagen density) with constant mechanical properties, and we indirectly evaluated the heterogeneity through the velocities measured in the experiments. Hence, the impact of this simplification in our results is quite low.

Nonetheless, in the future, we could achieve a better representation of the heterogeneity of collagen matrices through a model that might relate to a local collagen concentration with local viscosity values. For instance, further characterization experiments could be conducted to obtain more data, with which it would be possible to understand a more accurate description of this relationship. Moreover, it would be very useful to include the effects of matrix secretion and degradation by cells, which we have not considered in this first work. In fact, it is currently possible to simulate the cells’ capacity to secrete or degrade the surrounding ECM through some of PhysiCell’s built-in functions. Therefore, through this model, we could also study how the mechanical properties of the material are modulated by cells, as well as how this effect regulates cell migration and tumour formation.

Finally, due to the centre-based nature of the model, it was not possible to gain a realistic approximation of how cells may deform. However, for this particular study, we consider the decreased computational cost and the ability to simulate a high number of cells to be preferable to a detailed representation of cell geometry.

In conclusion, we have shown the model’s potential to simulate and describe the formation of tumour spheroids, integrating the biomechanical role of the matrix, which is mediated by its collagen concentration. As a consequence, our approach can qualitatively predict both individual cell behaviour in regard to cell motility and migration velocity, as well as pattern formation in spheroids, within different collagen densities. In the future, we will strive to characterize the local structural properties of the collagen hydrogels to create a more sophisticated model that can predict the trajectory of every single cell and, consequently, the formation of each spheroid. We expect that considering the real architecture of the matrix will provide more quantitative predictions, but it will require a high computational cost. Therefore, it is necessary to balance the potential benefits that a more accurate model can provide with the high experimental and computational cost that it requires.

## Supporting information

S1 Video Cluster growth through time. (GIF)

S1 Appendix Oxygen diffusion and consumption dynamics. (PDF)

S2 Appendix Model and data processing sensitivity analysis. (PDF)
