# Supplementary figures and images for: Extracellular matrix density regulates the formation of tumour spheroids through cell migration
Source: PLoS Comput Biol. 2021 Feb 26;17(2):e1008764. doi: 10.1371/journal.pcbi.1008764 (PMC7968691; doi:10.1371/journal.pcbi.1008764)

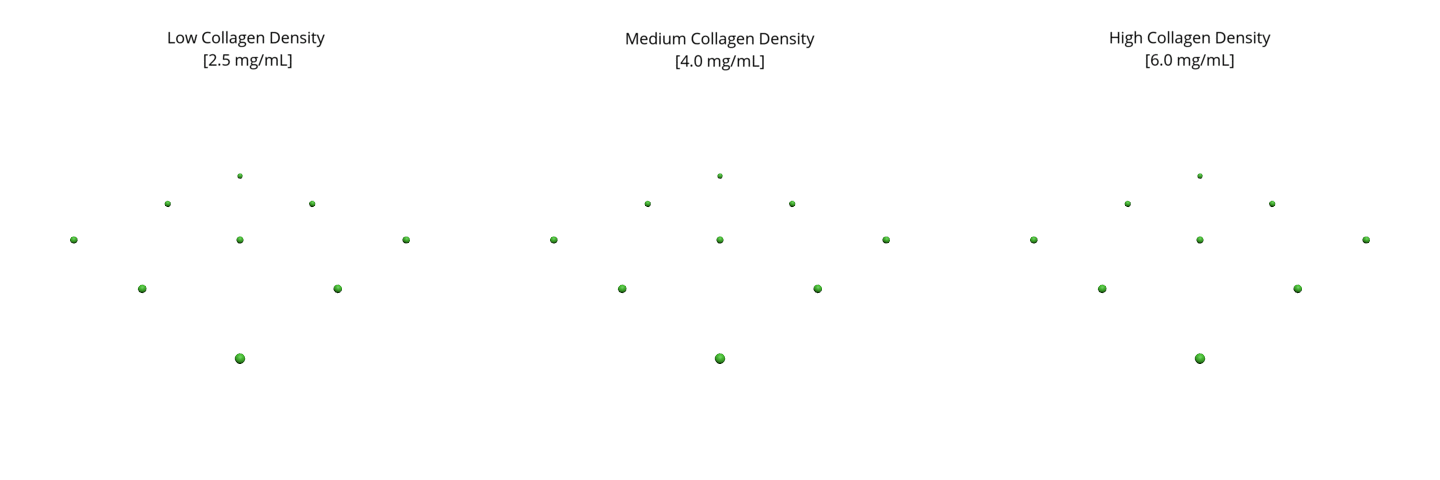

Supplement: S1 Video — (GIF) [file pcbi.1008764.s001.gif]
