# Supplementary material for: Extracellular matrix density regulates the formation of tumour spheroids through cell migration
Source: PLoS Comput Biol. 2021 Feb 26;17(2):e1008764. doi: 10.1371/journal.pcbi.1008764 (PMC7968691; doi:10.1371/journal.pcbi.1008764)
Supplement: S1 Appendix — (PDF) [file pcbi.1008764.s002.pdf]

# Supporting Information: Oxygen Consumption and Diffusion Dynamics

Although we have included oxygen and the effects that this substance may have on cells into our model, we do not consider it to play a significant role in our simulations. Considering the presence of oxygen and the cells' dependence on oxygen allows for a more accurate representation of cellular systems and can be useful to study other experimental configurations, to which our model can easily be adapted. Nevertheless, the experimental settings in which we base our simulations for this particular study are not expected to promote hypoxia, nor do we believe that changes in oxygen levels affected cell migration. Consequently, we do not consider oxygen to have a relevant effect on cell cycle or cell motility, either.

In this supplementary information document, we aim to present a study conducted with the simulated data for our model, to show that oxygen is present but does not play a significant role in our simulations. Hypoxia levels are defined in the model as oxygen pressure values below 15 mmHg, and cell necrosis starts occurring when pressure levels reach values lower than 5 mmHg. Furthermore, we set an initial oxygen concentration of 38 mmHg, which we expect to be lower than that found in experimental conditions.

To evaluate oxygen dynamics, we considered only the simulations that refer to multicellular cluster growth, as the oxygen consumption by a single cell in the individual cell motility study is not significant. We selected the middle plane (z=0) of the domain, as we expect this to be the plane with the highest cell density and, consequently, where oxygen consumption is maximal. Subsequently, we plotted a heatmap representing the oxygen concentration at each voxel for each of the studied collagen densities after five days of growth, as seen in Fig S1_1. From this data, it is possible to conclude that oxygen levels decrease over time, but never reach values lower than 30 mmHg.

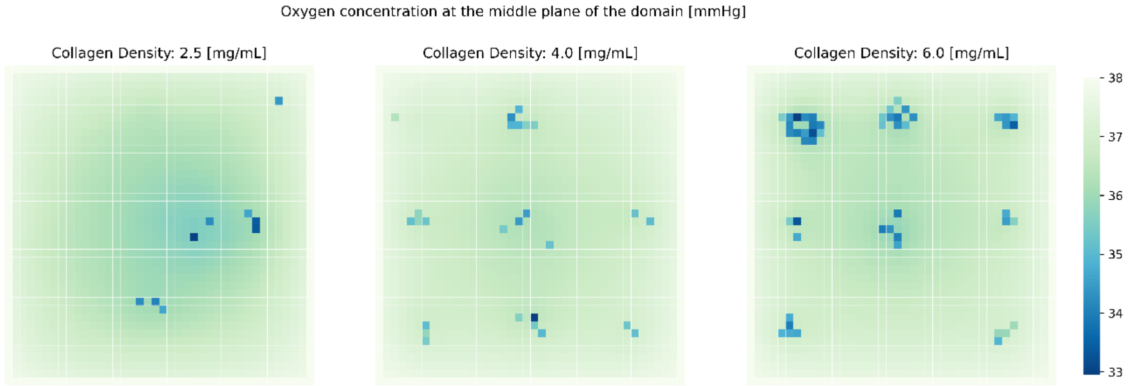


Fig S1_1. **Oxygen levels at 5 days of cluster growth**. Oxygen levels decrease over time and in response to differences in the matrix properties. Specifically, matrices of high density present lower oxygen levels, which can be attributed to the cells' restrained motility. As cells adopt a packed distribution, oxygen diffusion is hindered at the centre of the cell cluster. Contrarily, in low-density matrices, cells can migrate through the matrix and oxygen consumption does not overpower its diffusion. We note that this data corresponds to a single replicate, but it is representative of the results obtained in general. For a figure representing all replicates data, please see Fig S1_3.

For 7 days of growth, oxygen levels are shown to adopt lower values than those seen for 5 days, as expected. Nevertheless, hypoxia levels are not reached for this case either.

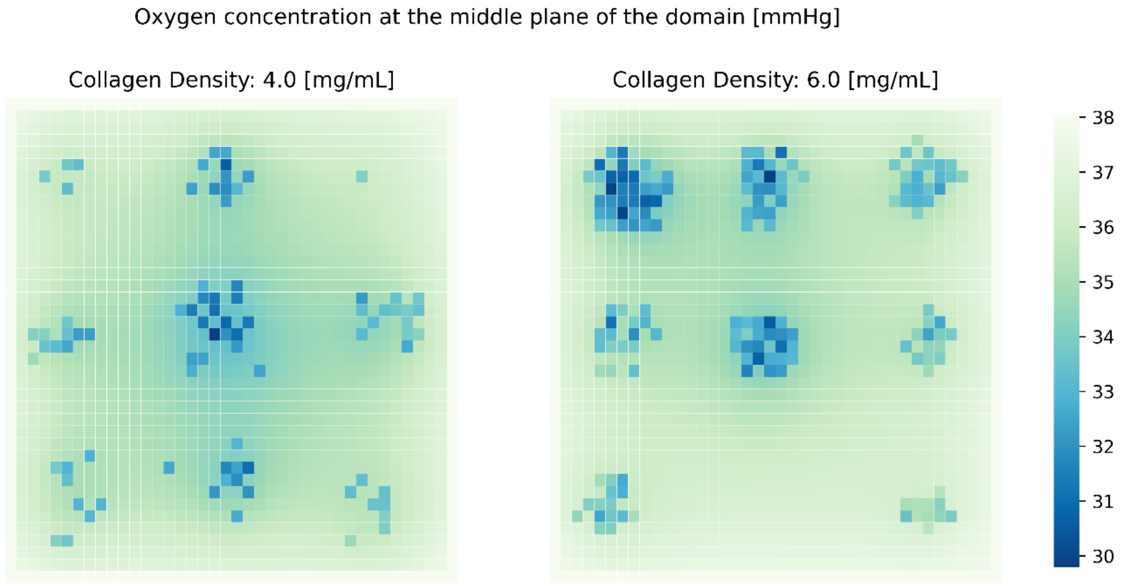


Fig S1_2. **Oxygen levels at 7 days of cluster growth**. Oxygen levels upon 7 days of growth follow the same trends as presented in Fig S1_1, yet are still always higher than the hypoxic threshold. Once again, this figure refers to a single replicate, yet it is coherent with our data.

Finally, for an extended quantitative analysis, Fig S1_3 showcases how the minimum oxygen concentrations at the middle plane change over time, for all 10 replicates. From this, we can conclude that the results are coherent throughout all of the replicates and that minimum values never adopt values lower than 29 mmHg, which is higher than the hypoxic threshold that activates cell necrosis and hindered cell proliferation.

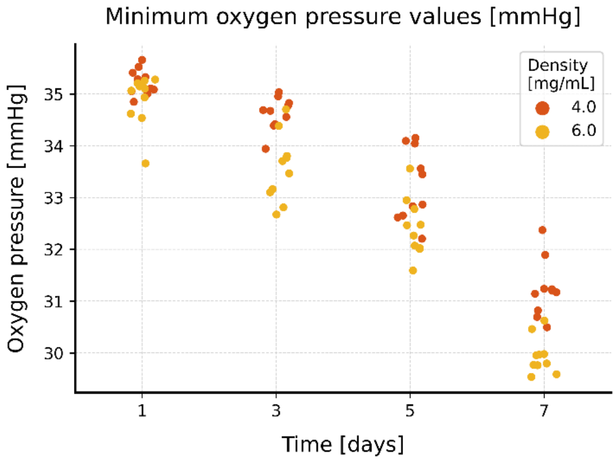


Fig S1_3. **Evolution of minimum oxygen levels through time**. We present a quantitative analysis of the minimum concentration values at the middle plane of the simulated domain. Our results show that the minimum values decrease through time but never reach pressure levels lower than 29 mmHg.

In conclusion, we infer that oxygen does not play a role in our simulations, due to our initial conditions, but its effect could be studied through our model, for different experimental configurations.
