# Supplementary material for: Extracellular matrix density regulates the formation of tumour spheroids through cell migration
Source: PLoS Comput Biol. 2021 Feb 26;17(2):e1008764. doi: 10.1371/journal.pcbi.1008764 (PMC7968691; doi:10.1371/journal.pcbi.1008764)
Supplement: S2 Appendix — (PDF) [file pcbi.1008764.s003.pdf]

Supporting Information: Model and Data Processing Sensitivity Analysis

The aim of our work consisted of qualitatively replicating the effect of collagen concentration on cell migration and multicellular cluster formation. Therefore, we have not conducted extensive studies to analyse how small perturbations in parameters influence our numerical results. Nonetheless, here we present a brief sensitivity analysis to confirm the robustness of our model and data processing methods, as well as the adequacy of our conclusions. We have chosen to focus on two main aspects of our work, which we consider to be the most significant to our results. Firstly, in regards to the model itself, we discuss how the choice of a locomotive force generator function influences the results for individual cell migration. Subsequently, we present a sensitivity analysis study on the parameters that define the algorithm used to classify cells into clusters.

Model Parameters

We have identified that the model parameters that play a significant role in our obtained results are related to the cell-generated locomotive forces. Furthermore, we have concluded that the cell-cell interaction parameters are not as relevant, and, thus, we have chosen values that are similar to those proposed by PhysiCell, published in previous works [53]- However, it must be remarked that the original values found in [53] were chosen to indirectly account for the extracellular matrix (ECM). Thus, since we have adapted this framework to directly account for the ECM, we have scaled the cell-cell interaction parameters accordingly, to avoid considering this effect twice. Taking this into consideration, we will mainly focus on how the function we have used to generate these cell-generated locomotive forces affects the model outputs.

Since our work regarded the qualitative trends observed experimentally, we have mostly focused on studying how different forces distributions influence our results, focusing on the shape of the distributions. Based on the assumption that the main factors acting on cell velocity (in the single-cell setup) are the cell-generated locomotive forces, as well as the drag forces determined by the ECM, and to avoid the computational cost of the model, we have built a simplified script to study the effect of these forces. In particular, we have built a Python script that implements the equation below (based on Eq 4 in our manuscript), taking into account values from a given force distribution and the effect of the ECM, through the dynamic viscosity of the matrix.

$$\mathrm{v}_i = \frac{1}{\eta}\left(\mathrm{F}_{loc}^{i}\right)$$

Here, F_{loc}^{i} is a value chosen at random from the chosen distribution and η is the dynamic viscosity of the collagen matrix. For this sensitivity study, we have considered a normal distribution with a mean value, μ, of 0.15 and a standard deviation, σ, of 0.2; a normal distribution with $\mu = 0.0$, $\sigma = 0.45$; and a lognormal distribution with $\mu = 0.0$, $\sigma = 0.7$. The parameters of each function were fitted to provide the best possible results, and we compiled a number of velocity values comparable to that measured experimentally. The results

obtained for this study are summarized in Fig S2_1, from which we conclude that the shape of the chosen distribution is highly significant and can lead to different cell behaviours.

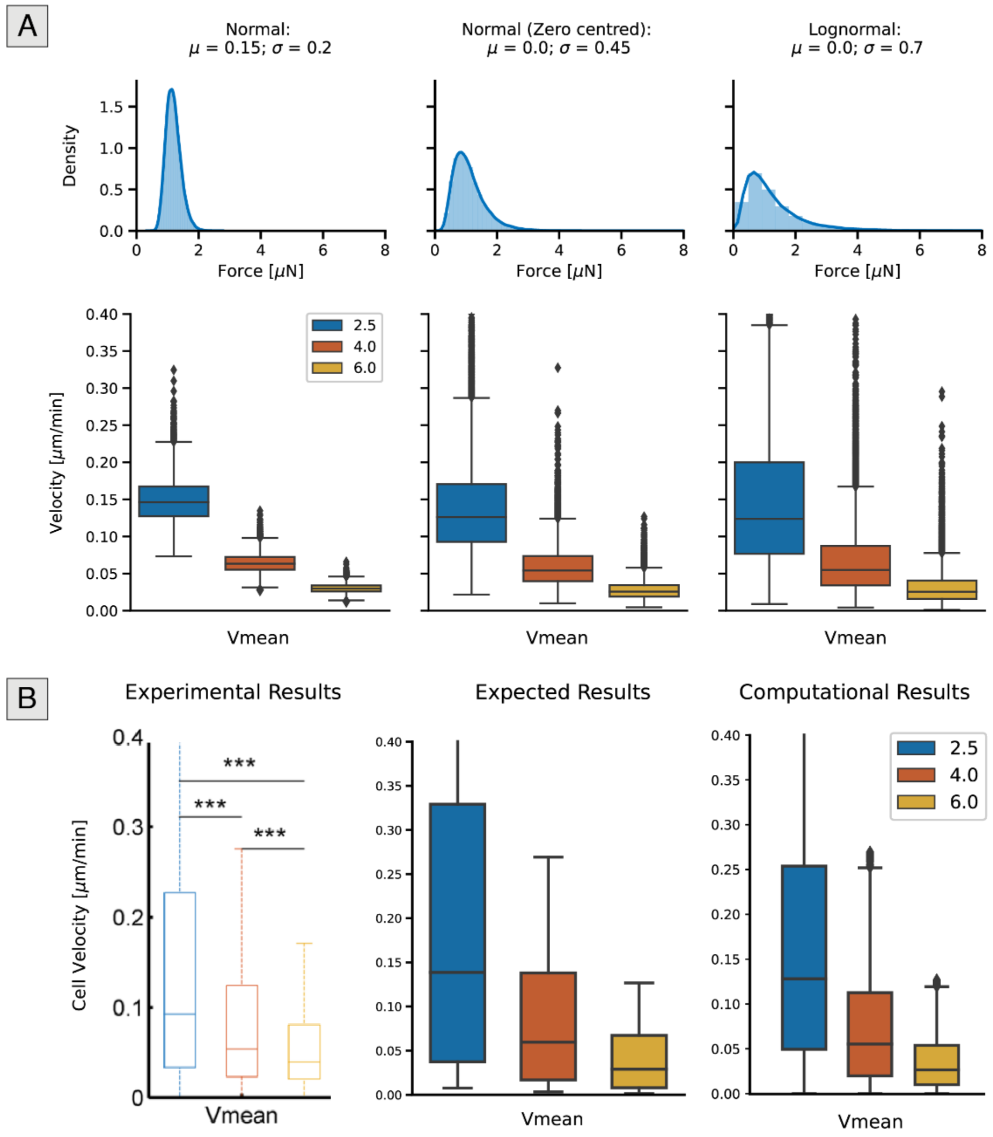

Fig S2_1. **Estimated results for cell velocities in function of different force generator functions**. (A) Estimated velocity distributions for a normal distribution, a normal distribution centred at zero and a lognormal distribution (top). To study how the force generator influences the computational results, we have studied multiple force distribution functions and their effect on cell velocity. It can be concluded that, despite capturing the median velocities, these distributions fail to replicate the range of the experimental values, as well as the lack of outliers. Hence, we conclude that the chosen distribution highly impacts the obtained results. (B) Comparison between the experimental results (left), the simplified model (centre) and the computational results obtained with the actual model (right). Although the simplified model predicted a broader distribution for cells grown in low-density

matrices, we consider that the conclusions obtained with this implementation apply to our proposed model.

Extending this analysis, we have also studied how the coefficients of our force generator function may influence our results. For our implementation, which was based on the empirical velocity distributions and fitted accordingly, the velocity values depend on the coefficients of a function given by a general third-degree polynomial form

$$y(x) = ax^3 + bx^2 + cx + d$$

which we have fitted to become

$$F_{loc}(x) = 1.56x^3 + 3.27x^2 + 0.07x + 0.06$$

Particularly, we have studied how these coefficients influence the mean, median and maximum cell velocity values. We have chosen to use the cells grown in medium-density matrices as an example since the viscosity of the matrix affects all these parameters equally, and, thus, we expect the changes to be comparable between matrix densities. The results of this study are summarized in Fig S2_2. Based on this figure, we conclude that our model is robust to small variations in these coefficients.

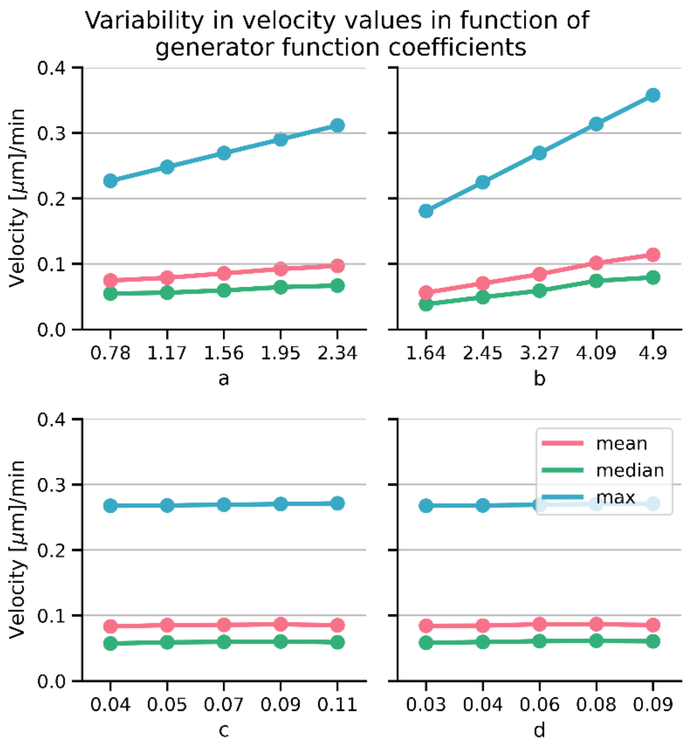

Fig S2_2. **Effect of the generator function coefficients on the cell velocity results**. Mean (red), median (green) and maximum (blue) values for the instantaneous cell velocities of cells grown in medium-density matrices, for different parameter values. For each plot, a single parameter was changed, while the others were kept at the values presented in the paper (a=1.56, b=3.27, c=0.07, d=0.06). The coefficients were varied based on their magnitude. For small changes, especially those regarding coefficients c and d, the velocity values do not appear to be largely affected. However, for more significant increases both in a and b, the velocities values increase with these parameters, in particular the maximum velocity value.

Data Analysis Parameters

Apart from the model parameters, some aspects of the data processing methodology used may also influence our results, particularly for the multicellular setup. In order to quantify the area and eccentricity of the multicellular clusters observed after some days of growth, cells must be classified into clusters. Although we have tried to replicate the type of processing used in the experimental results, this was not completely possible, as there are some differences the experimental and image-based data and our computational results, that are based on the coordinates of the centres of the cells. Henceforth, some parameters had to be chosen and fitted, namely the radius and the minimum number of cells considered by the clustering algorithm, as well as the height of interest that we have defined to replicate the effect of an image-based analysis.

To classify cells into clusters, we have chosen to select a defined region of the z-axis, to simulate the effect of the microscopy-based analysis, for which cells that are in different planes appear out of focus and are disregarded. Therefore, we have aimed to replicate this effect by selecting a smaller region and removing all cells whose centres are not in the said area. Fig S2_3 shows how the area results change with this parameter. Based on these results, we have selected a height of interest of 48 μm, corresponding to the diameter of two cells, to avoid misclassifying cells that are in different planes and that do not belong to the main clusters.

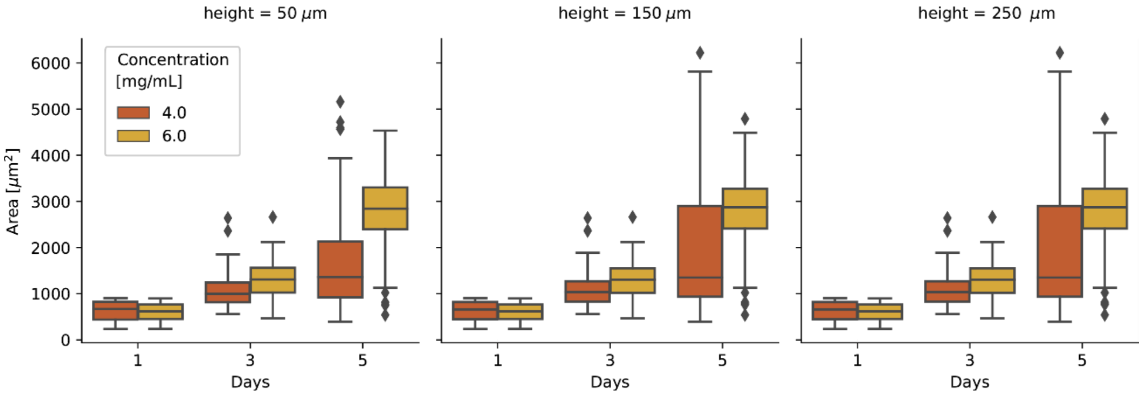

Fig S2_3. **Effect of the height of interest on cluster area.** Distributions of the cluster area through time, in function of the selected height of interest, for high (represented in yellow) and medium (represented in orange) collagen densities. The value of the height parameter mainly affects the results obtained for cells grown in medium collagen densities, as these present enhanced motility abilities. Accordingly, cells are more likely to move through different planes of the z-axis and appear to be part of some clusters when seen in 2D. Yet, they are, in fact, on different planes and, consequently, do not belong to these structures. With this in mind, we selected a value of 48, three times the diameter of a cell, which reduces the number of these outliers.

We have chosen to classify cells into clusters through the implementation of the density-based spatial clustering of applications with noise (DBSCAN) [59,60], which requires the user to define the radius of interaction and the minimum number of cells in a cluster. For these parameters, we have aimed to choose a radius of interaction that was slightly larger than the radius of two cells, so that only cells that were close to each other were selected.

Moreover, we initially defined that the minimum of clusters of cells in the radius of interaction should be 3. However, given that the experimental results suggest that there are clusters at day 1, and cells are only able to replicate once in that period, we have defined that, at day 1, clusters could be composed by a minimum number of cells of 2. We have only used this value for day 1, though, as we have observed that this also promoted the classification of single cells into clusters. More information on the effect of these parameters can be found in Fig S2_4.

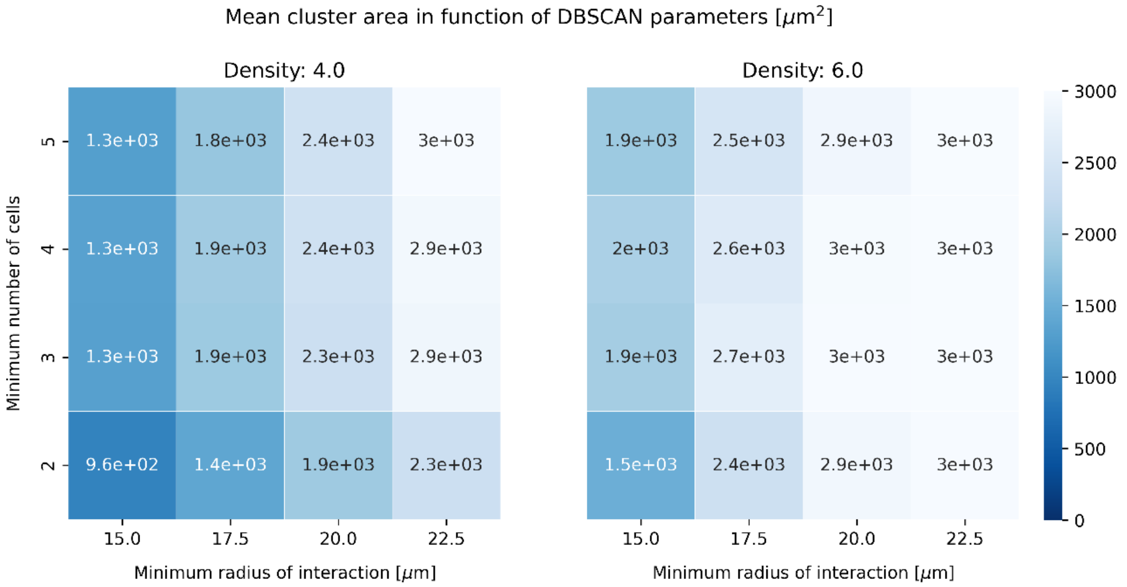

Fig S2_4. **Effect of the DBSCAN parameters on cluster area values.** Mean cluster area for different values of the radius of interaction and the minimum number of cells in said radius, at day 5. On the one hand, the effect of different values of the minimum number of cells is mainly noticeable between 2 and 3. A smaller minimum number of cells leads to the detection of small aggregates, that do not truly classify as clusters, reducing the value of the mean cluster area. On the other hand, the value of the radius of interaction appears to have a more significant effect, particularly for clusters grown in medium-density matrices. This is probably explained by having more cells scattered through the domain, which may be detected through an increase in the radius magnitude. Contrarily, high-density matrices produce large clusters that are fairly distanced from each other and do not present individual cells.

Regarding the eccentricity values, the state of development of the tumour at seven days of growth allows for the clusters to be more defined, as cells grow and connect, forming large tumours. Hence, we have concluded that the height of interest no longer plays a significant role at this stage of growth, as presented in Fig S2_5.

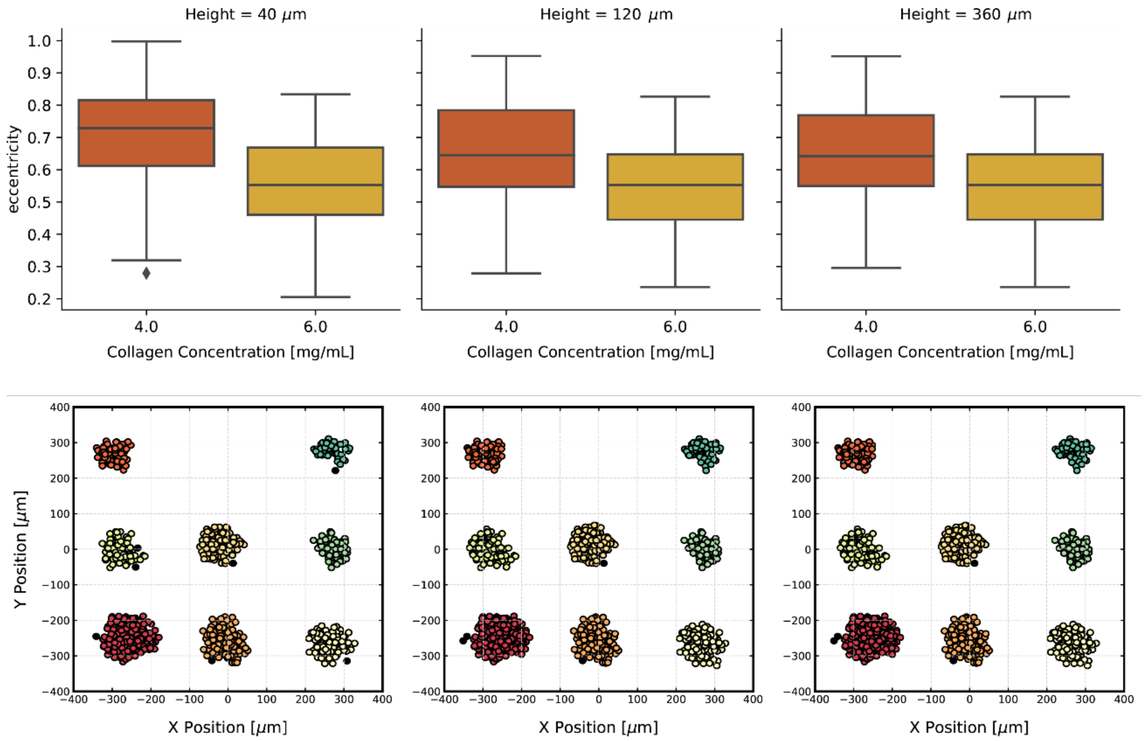

Fig S2_5. **Effect of the height of interest on cluster eccentricity.** Due to the state of development of the tumour clusters after 7 days of growth, associated with the decreased motility observed in medium and high collagen densities, the clusters are large and well-defined, as almost all cells contact with each other. Moreover, there are barely any individual cells that do not belong to one of the clusters. Therefore, it becomes less relevant to limit the area of interest, to avoid cells that may be on top of the tumour, but not connected to the structure.

Taking this into consideration, we have chosen to consider the entire computational domain to study cluster eccentricity. Moreover, we have used the same parameter values for our cluster area study, as we have observed that changes in these parameters would not lead to significant differences in eccentricity values, as presented in Fig S2_6.

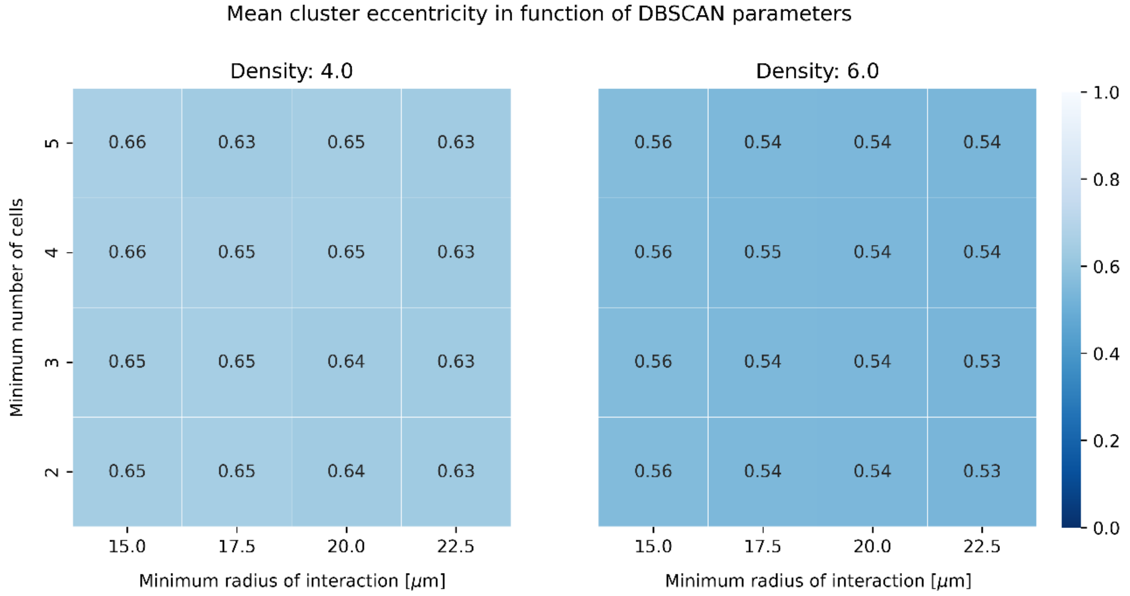

Fig S2_6. **Effect of the DBSCAN parameters on cluster eccentricity values**. For a defined height of 300 (the entirety of the domain), changes in the DBSCAN parameters do not produce significant changes in the computed eccentricity values. Therefore, we opted to keep the same values as those used to compute cluster area, to keep our methods consistent.

Finally, we would like to comment on the fact that cluster eccentricity values are highly sensitive to small variations in cluster dimensions. Accordingly, although the computational results are robust to small perturbations in parameter values, there are some differences between the experimental and computational results, as can be seen in Fig S2_7. In particular, we have observed that it was very difficult to obtain eccentricity values as low as those seen experimentally.

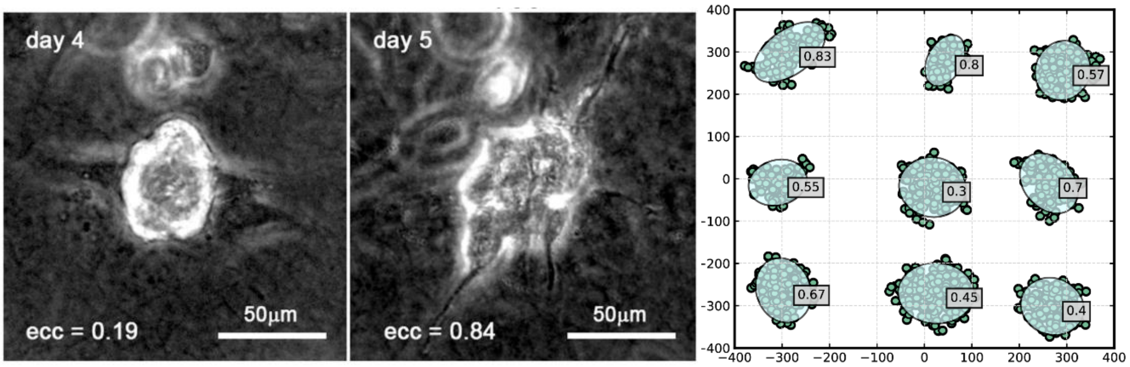

Fig S2_7. **Differences between the experimental and computational values for cluster eccentricity**. Experimental (left) and computational (right) results for cluster eccentricity (the computational results also present the equivalent ellipse from which these values have been calculated). It must be noted that an eccentricity of zero indicates a round cluster, whereas an eccentricity of one indicates a cluster that resembles a line. The experimental and computational results present differences in cluster eccentricity, although a visual analysis may suggest comparable eccentricity values. For instance, the centre cluster in the computational dataset presents an eccentricity of 0.3. Compared to the experimental results for day 4, it seems to be as round, if not more, than the experimental cluster. Yet, the eccentricity value of the latter is of 0.19. Similarly, we observed this pattern for several of the computational clusters.

Cluster eccentricity is computed based on the dimensions of the ellipse that best fits the cluster. In particular, taking an ellipse's major, a, and minor, b, axis, we compute its eccentricity using the following equation

$$eccentricity = \frac{\sqrt{a^2 - b^2}}{a^2}$$

Therefore, we can plot cluster eccentricity in regards to the ratio between a and b, as presented in Fig S2_8. This plot allows us to confirm that cluster eccentricity is particularly sensitive at low eccentricity values. In this range (between 0-0.3, approximately), very small differences between the length of the ellipse axes greatly influence the eccentricity values. We note that our computational data is very sensitive to these differences, which are in the order of <10 μm, as we can keep track of the coordinates of each cell. However, the experimental results, which are image-based, present a lower sensitivity, as they are limited by the size of each pixel. Henceforth, experimental results present lower eccentricity values.

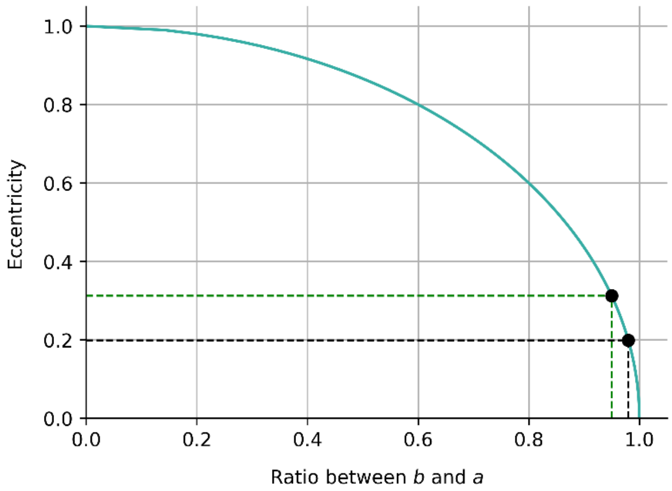

Fig S2_8. **Effect of the ratio between the major and minor axes of an ellipse on cluster eccentricity.** The relationship between ellipse eccentricity and the ratio between its major and minor axes dictates that, for low eccentricity values, the outcome is highly sensitive to differences in the dimensions of the ellipse. For a cluster with a major axis of 100 µm, a difference of just 5% between *a* and *b* (which, in this case, is just around 5 µm) produces an eccentricity of around 0.3 (green lines). Furthermore, a difference of just 2 µm between the two axes of this cluster produces an eccentricity value of 0.2 (black lines). Contrarily, as the eccentricity values increase, the output becomes less sensitive to these differences.
